# Construction and Transcriptomic Study of Chicken IFNAR1-Knockout Cell Line Reveals the Essential Roles of Cell Growth- and Apoptosis-Related Pathways in Duck Tembusu Virus Infection

**DOI:** 10.3390/v14102225

**Published:** 2022-10-09

**Authors:** Chengwei Xiang, Zekun Yang, Ting Xiong, Ting Wang, Jie Yang, Mei Huang, Dingxiang Liu, RuiAi Chen

**Affiliations:** 1 College of Veterinary Medicine, South China Agricultural University, Guangzhou 510642, China; 2 Zhaoqing Branch Center of Guangdong Laboratory for Lingnan Modern Agricultural Science and Technology, Zhaoqing 526000, China; 3 Zhaoqing Institute of Biotechnology Co., Ltd., Zhaoqing 526238, China; 4 Integrative Microbiology Research Centre, South China Agricultural University, Guangzhou 510642, China

**Keywords:** DF-1, CRISPR/Cas9, IFNAR1, duck Tembusu virus, transcriptomic analysis

## Abstract

For industrial vaccine production, overwhelming the existing antiviral innate immune response dominated by type I interferons (IFN-I) in cells would be a key factor improving the effectiveness and production cost of vaccines. In this study, we report the construction of an IFN-I receptor 1 (IFNAR1)-knockout DF-1 cell line (KO-IFNAR1), which supports much more efficient replication of the duck Tembusu virus (DTMUV), Newcastle disease virus (NDV) and gammacoronavirus infectious bronchitis virus (IBV). Transcriptomic analysis of DTMUV-infected KO-IFNAR1 cells demonstrated that DTMUV mainly activated genes and signaling pathways related to cell growth and apoptosis. Among them, JUN, MYC and NFKBIA were significantly up-regulated. Furthermore, knockdown of zinc-fingered helicase 2 (HELZ2) and interferon-α-inducible protein 6 (IFI6), the two genes up-regulated in both wild type and KO-IFNAR1 cells, significantly increased the replication of DTMUV RNA. This study paves the way for further studying the mechanism underlying the DTMUV-mediated IFN-I-independent regulation of virus replication, and meanwhile provides a potential cell resource for efficient production of cell-based avian virus vaccines.

## 1. Introduction

Type I interferons (IFN-I) play essential roles in cellular responses to virus infection. Upon binding to the type I IFN receptor (IFNAR), IFN-I activates the Janus kinase (JAK)-signal transducer and activator of transcription (STAT) signaling pathway and induces the expression of IFN-stimulated genes (ISGs) [1]. IFNAR consists of IFNAR1 and IFNAR2, each forms heterodimers composed of α and β subunits. JAK-STAT pathway is one of the multiple pathways that are involved in transmitting the signaling from the IFNAR complex to the nucleus [2]. In this signaling pathway, IFNAR1 binds to tyrosine kinase 2 (Tyk2), while IFNAR2 binds to Jak1, working together to activate the STAT transcription factors [3] and ultimately mediate the production of ISGs (MX, OASL, VIPERIN, IFIT5, and many more) to establish an antiviral state in the cell.

Virus infection of cells induces the expression of a diversity of host gene expression, either dependent or independent of IFNs. In cells infected with duck Tembusu virus (DTMUV), IFN regulatory factor 1 (IRF1) was previously shown to be significantly up-regulated [4]. IRF1 can directly bind to the promoters of several ISGs, including VIPERIN, IFIT5 and CMPK2, to activate their transcription and inhibit DTMUV replication [4]. In addition to inducing the expression of IFN-I and IFN-I-dependent ISGs, IRF1 may also mediate the expression of host genes via an IFN-I-independent mechanism to suppress the replication of DTMUV [4]. As IFNAR1 plays an important role in initiating the IFN-I response, this possibility was explored by construction of a chicken IFNAR1 (chIFNAR1)-knockout DF-1 cell line (KO-IFNAR1) using the CRISPR/Cas9 gene editing technique, establishing the functional role of IRF1 in IFN-I-dependent and independent anti-DTMUV responses.

Viruses utilize different strategies to either antagonize or agonize the physiological functions of normal cellular signaling pathways and processes for efficiently replicating and completing their infectious cycle. For example, activation of the mitogen-activated protein kinase (MAPK) pathway has been observed during different viral infections, regulating various aspects of virus–host interactions. MAPKs are conserved kinases that regulate key cellular signaling pathways, such as apoptosis, differentiation, and immune responses [5]. Infection of chicken lines by highly pathogenic avian influenza virus (HPAIV) significantly up-regulates the signaling molecules of the MAPK pathway, including AP-1, c-FOS, JUN and c-Myc, suggesting that the antiviral activity of HPAIV-resistant chicken lines was mainly mediated by the MAPK signaling pathway. Most AP-1 transcription factors are also up-regulated during coronavirus infection of cultured cells and chicken embryos, and up-regulation of cFOS at early to intermediate phases of the viral replication cycle promotes viral replication by the suppression of apoptosis [6,7].

The CRISPR/cas9 system includes a Cas9 protein with endonuclease activity and a single-stranded guide RNA (sgRNA). The sgRNAs bind to the Cas9 protein, and they are then targeted to designated sites on the genomic DNA duplex, achieving site-directed cleavage, resulting in double strand breaks (DSB) [8,9]. After cleavage, cells can use both the nonhomologous end joining (NHEJ) and homology Directed Repair (HDR) mechanisms to repair the broken DNA double-strand [10]. Therefore, the CRISPR/Cas9 gene editing technology can cause the deletion or insertion of bases at the target site, resulting in the loss of gene function. The construction of gene function-deficient cell lines has guiding significance for the study of gene function.

In this study, the effects of chIFNAR1-knockout on the replication of duck Tembusu virus (DTMUV), Newcastle disease virus (NDV) and gammacoronavirus infectious bronchitis virus (IBV) were systematically investigated, showing markedly more efficient viral replication in the knockout cells. Transcriptome sequencing of DTMU-infected wild type and KO-IFNAR1 cells further revealed the expression profiles of DTMUV-induced innate immune system-related genes and mechanisms underlying the IFN-I-independent regulation of DTMUV replication.

## 2. Materials and Methods

### 2.1. Cell Culture

DF-1 cells (chicken fibroblast cell line) were cultured at 37 °C with 5% CO_2_ in DMEM supplemented with 10% fetal bovine serum (FBS), 100 U/mL penicillin and 100 μg/mL streptomycin.

### 2.2. Construction of chIFNAR1-Knockout DF-1 Cell Clone, KO-IFNAR1

Targeting strategy: based on the chIFNAR1 sequence obtained from the NCBI database, two gRNA sequences targeting CDS2 of chIFNAR1, sgRNA1 (ACCCTAATGTGGAACTACAC) and sgRNA2 (TTGTGCTGAAAATGTCACGT), were designed, synthesized and cloned into pX459-Cas9 vector under the control of U6 promoter, yielding two sgRNA expression plasmids, pX459-sgIFNAR1-1 and pX459-sgIFNAR1-2, respectively.

Cell transfection and drug selection: transfection of plasmid DNA was performed using TransIntroTM EL transfection reagent (Transgen, Beijing, China) according to the manufacturer’s instructions. Briefly, cells were seeded in 12-well plates the day before transfection. For each transfection, 1.6 µg pX459-sgIFNAR1 and 3 µL TransIntroTM EL were diluted in 100 µL normal medium, incubated for 5 min and mixed by gentle pipetting. After an additional 20 min incubation, the medium was replaced with 800 µL FBS-free DMEM and the transfection mix was added. Cells were incubated at 37 °C for 4–6 h before the medium was replaced with the complete medium. After a 48-h recovery period, cells were supplemented with 5 µg/mL puromycin (Sigma, New York, USA) in the medium for 14 days until successful selection.

Screening of single clones and sequencing of the genomic DNA: single colonies were picked, transferred to a new 35 mm culture dish per clone and cultured until confluence. Cells from each dish were collected for PCR analysis using TAKARA’s Premix Taq with primer pair GCTTACAATCACCCGTGGCA and ACCAGTTACACTCGTGCTGG. The purified PCR products were cloned and sent to Shanghai Sangon Biological Co., Ltd., for sequencing.

### 2.3. CCK8 Assay

The proliferation of DF-1 and KO-IFNAR1 cells was assessed by the Cell Counting Kit-8 (CCK8) assay. Briefly, cells were seeded individually in 96-well plates (4000 cells/well) and 10 µL of CCK8 reagent was added. Signals were measured at an absorption wavelength of 450 nm at 12, 24, 36, 48, 72 and 96 h post-CCK8 treatment, respectively.

### 2.4. RNA Extraction and RT-qPCR Analysis

Total RNA was extracted using the TRIzol reagent (Invitrogen, Carlsbad, CA, USA), reverse-transcribed using the PrimeScript^TM^ RT Master Mix (Takara, Kusatsu, Japan). Total RNA was extracted by Trizol method as follows: DF-1 cells and DF-1-IFNAR1-/- cells infected with DTMUV, NDV and IBV were harvested at 8, 12, 24 and 36 h post-infection, respectively. After freeze-thawing 3 times, 250 μL of virus solution was added to 750 μL of Trizol, shaken well for 30 sec, and placed at room temperature for 5 min. Next, 200 μL of chloroform was added, mixed well, and left at room temperature for 2 min. The above mixture was centrifuged at 4 °C for 10 min at 12,000× *g*. Then, about 500 μL of the supernatant was aspirated, an equal volume of isopropanol was added, inverted and mix, and placed at −20 °C for 30 min or overnight. The above mixture was centrifuged again at 4 °C at 12,000× *g* for 10 min. The supernatant was discarded, then 1 mL of 70% DEPC ethanol was added to wash once, centrifuged at 4 °C, and centrifuged at 12,000× *g* for 5 min. Next, the liquid was discarded, the remaining liquid was sucked up with the pipette tip, and the collected sediment was fully dried in the ultra-clean bench for about 10 min. Then, 20 μL of DEPC water was added to dissolve, pipetted several times with a pipette tip, and placed in a dry bath at 60 °C for 10 min to promote complete dissolution. Genomic DNA was then removed by using DNase I (TaKara, Kusatsu, Japan). Reverse transcription was performed after the RNA concentration was determined by a nucleic acid analyzer. RNA levels of each transcript were determined by quantitative PCR (qPCR) with the SYBR^®^ Premix Ex Taq^TM^ II Kid according to the manufacturer’s instructions. The qPCR analysis was performed using a QuantStudio 3 Real-Time PCR System (Applied Biosystems, Waltham, MA, USA), and the relative abundance of each transcript was calculated using the 2^−^^ΔΔCT^ method after normalizing to the internal β-actin value. The gene specific primers for qPCR are shown in Table 1.

### 2.5. SDS-PAGE and Western Blot Analysis

Cells were harvested at 8, 12, 24, 36 and 48 h post-infection (hpi), respectively, and lysates were prepared. Cell lysates (25 µg protein) were mixed with 5 × LaemmLi sample buffer, incubated at 90 °C for 5 min and analyzed in sodium dodecyl sulfate-12% polyacrylamide gel using the Bio-Rad Mini-PROT EAN Tetra Cell System (Purchased from BIO RAD, New York, NY, USA). The separated proteins were transferred to nitrocellulose membranes using the Bio-Rad TransBlot Protein Transfer System. Antibodies against β-actin (#4967) and FLAG-tag (#2044) were purchased from Cell Signaling Technology (Danvers, MA, USA). Monoclonal antibodies against DTMUV E protein, NDV HN protein and IBV N protein were prepared and provided by the Laboratory of Infectious Diseases, School of Veterinary Medicine, South China Agricultural University. After incubation with 1 µg/mL primary antibodies against NDV HN, DTMUV E and IBV N overnight at 4 °C, membranes were incubated with 1:15,000 diluted fluorescein isothiocyanate -labeled goat anti-mouse IgG or goat anti-rabbit IgG for 1 h at room temperature, and proteins were detected with an Azure c600 imager according to the manufacturer’s instructions. Proteins were quantified by densitometry using NIH software Image J (https://imagej.nih.gov/ij/) (accessed on 9 July 2021). All experiments were repeated three times with similar results and a representative result is shown.

### 2.6. Transcriptomic Analysis

Transcriptomic analysis was performed by Majorbio Technologies Co., Ltd., Shanghai, China. Cells were grown on a 10 cm diameter plate and rinsed twice with serum-free medium before undergoing infection with DTMUV at a multiplicity of infection (MOI) of 1 in serum-free medium. In mock controls, identical amounts of UV-inactivated DTMUV were utilized. Total RNAs were prepared from DTMUV-infected DF-1 and KO-IFNAR1 cells harvested at 24 hpi, and sequenced using the Illumina HiSeq sequencing technology platform to construct a transcriptome library and obtain sequencing data.

To identify differentially expressed genes (DEGs) between DTMUV-infected and mock-treated DF-1 and KO-IFNAR1 samples, the expression levels of each transcript were mapped according to the number of fragments per million exons per kilobase (FRKM) method. RSEM 4 [11] was used to quantify gene abundance, and the R statistical package Empirical Analysis of Digital Gene Expression in R (EdgeR) 5 [12] was used for differential expression analysis. In addition, functional enrichment analysis, including GO and KEGG, was performed to determine which DEGs were significantly enriched in GO terms and metabolic pathways with Bonferroni-corrected *p*-value ≤ 0.05 compared to the transcriptome-wide background. GO functional enrichment and KEGG pathway analysis were performed by Goatools 6 [13].

Gene co-expression networks were constructed using the R package WGCNA 1.51 [14]. After screening, 100 DEGs from 4 samples were introduced into WGCNA to construct co-expression modules. The expression correlation coefficients of DEGs are then calculated to select an appropriate soft threshold and finally achieve a scale-free topology. A network dendrogram was built using average link hierarchical clustering of topologically overlapping distinct similarity matrices (1-TOM). DEGs with similar expression patterns were classified according to TOM differences. The minimum module size is 30, and the merging threshold for partial module combinations is set to 0.25. To identify biologically meaningful modules, module eigengenes were used to calculate correlation coefficients. The intra-module connections (functional soft connections) were calculated for each DEG, and the networks were visualized using Cytoscape v3.3.0 [15].

### 2.7. RNA Interference

Chicken zinc-fingered helicase 2 (HELZ2) siRNA (+): 5’-GCUGUUCCUUGAGGAUUATT-3’, siRNA (-): 5’-UAAUCCUCAAGGGAACAGCTT -3’, chicken interferon-α-inducible protein 6 (IFI6) siRNA (+): 5’-CCACAAA GCCGGUUUCACUTT-3’, siRNA (-): 5’ -AGUGAAACCGGCUUUGUGGTT-3’ and NC siRNA (+): 5’-UUCUCCGAACGUGUCACGUTT-3’, siRNA (-): 5’-ACG UGAC ACGUUCGGAGAATT were purchased from Sangon Biotech (Shanghai, China). Transfection of siRNA was performed using TransIntro™ EL Transfection Reagent(TransGen Biotech, Beijing, China) according to the manufacturer’s instructions. Five hours after transfection, cells were infected with DTMUV at an MOI of 1 or mock-treated, and incubated until harvest at 24, 36 and 48 hpi, respectively.

### 2.8. Plasmid Construction

Expression plasmid XJ40-FLAG-chIFNAR1 was generated by introducing the relevant PCR products to a pXJ40-based plasmid. The PCR products were amplified with primer pairs: chicken IFNAR1, 5′ -GGATCCAATGGCTGAGGCGGCGTGTG-3′, and 5′ CTCGATTATAGTATGTCATTTCCTAGTGTTTCTTCTGACCCT-3′.

## 3. Results

### 3.1. CRISPR/Cas9-Mediated Knockout of chIFNAR1 in DF-1 Cells

**Figure 1:**
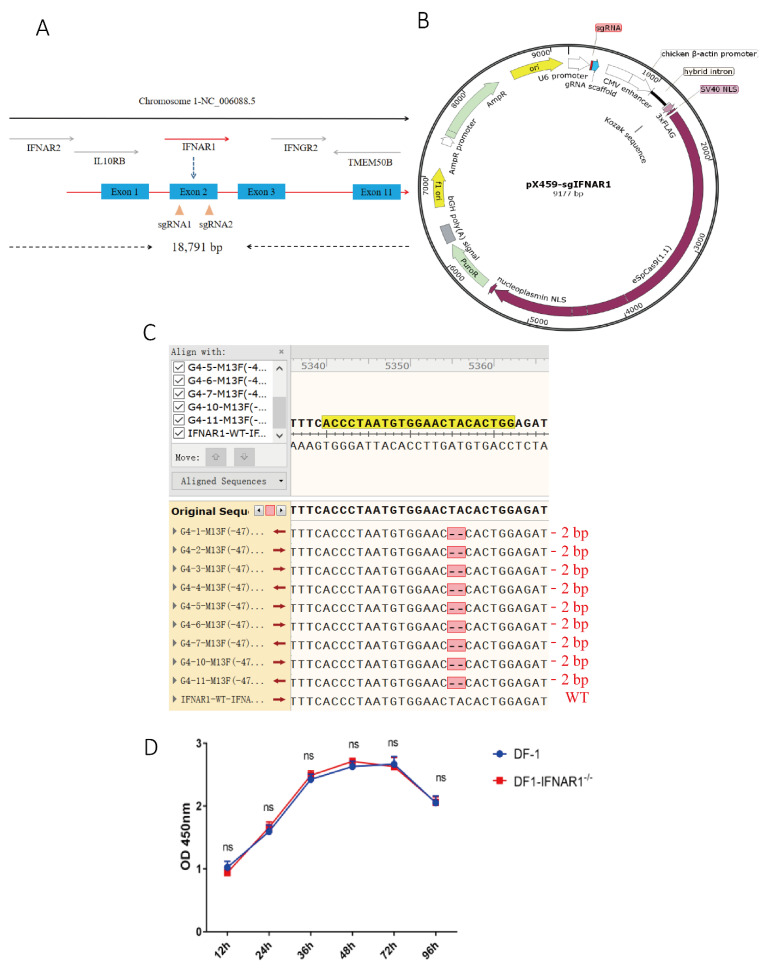
Construction of KO-IFNAR1 cell line. (**A**) Schematic diagram of chIFNAR1 gene and target sites. The two target sites were designed based on the sequence of exon 1. (**B**) Schematic diagram of the pX459 vector used for gene knockout in this study. (**C**) Sequencing results of targeting regions in KO-IFNAR1 monoclonal cells. Sequence alignment in-between revealed a homozygous 2 bp-deletion in KO-IFNAR1 cells. (**D**) Proliferation of KO-IFNAR1 cell line using CCK8 assay. ns, non-significant.

Sequence analysis showed that the chIFNAR1 gene was located on chromosome 1, containing 18,791 base pairs and 11 exons (Figure 1A). Two sgRNA unique sequences targeting different positions of chIFNAR1 exon 2 were cloned downstream of the U6 promoter in pX459 (Figure 1B), generating pX459-sgIFNAR1-1 and pX459-sgIFNAR1-2 (Figure S1). The two plasmids were transfected into DF-1 cells, respectively, and screened with puromycin. Sequencing analysis showed abnormal peaks at the sgRNA-targeting regions, confirming that both sgRNAs can be effectively used to knock out the IFNAR1 gene (Figure S2), and sgRNA1 was used in further experiments.

Cells transfected with IFNAR1-sgRNA1 were screened for a monoclonal cell line deficient in the chIFNAR1 gene, using the limiting dilution method. Ten monoclonal cell lines were selected and DNA sequencing of the IFNAR1 gene showed a homozygous mutation in KO-IFNAR1 cells. Nine out of the ten monoclonal cell clones contain a deletion of two bases at positions 232 (T) and 233 (A), which would result in the early termination of protein translation at the 52nd amino acid (N) Figure 1C and Figure S3).

The proliferation of wild type DF-1 and KO-IFNAR1 cells was assessed by measuring the absorbance at 450 nm using the CCK-8 method, showing that knockout of the IFNAR1 gene did not affect the proliferation of KO-IFNAR1 cells. This result confirms the successful construction of a stably passaged, IFNAR1-knockout chicken cell line, which was used in the subsequent experiments (Figure 1D).

### 3.2. Knockout of IFNAR1 Significantly Reduces the Induction of IFN-β and ISG in Cells Infected with DTMUV, IBV and NDV

**Figure 2:**
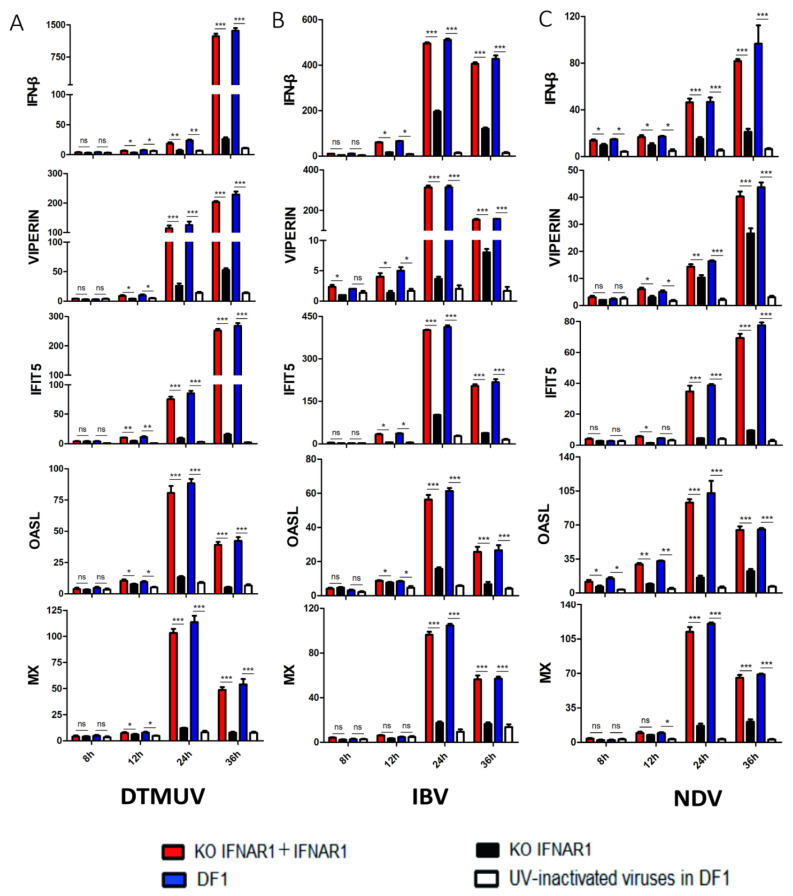
Suppression of ISG induction in IFNAR1-knockout cells infected with DTMUV, IBV and NDV, respectively. The mRNA levels of IFN-β, VIPERN, IFIT5, OASL and MX in cells infected with DTMUV (**A**), IBV (**B**) and NDV (**C**) were detected by RT-qPCR. T tests were conducted between the FINAR1 overexpressed cells (red) and mock cells in KO-IFNAR1 (black), and were conducted between the wild type DF-1 cells (blue) and UV-inactivated viruses treated DF-1 cells (white). * *p* < 0.05; ** *p* < 0.01; *** *p* < 0.001; ns, non-significant.

The effect of IFNAR1-knockout on the induction of ISGs by virus infection was then carried out. In wild type DF-1 cells infected with DTMUV, IFN-β and four common ISGs, IFIT5, VIPERIN, OASL and MX, were greatly induced in wild type DF-1 cells, induction levels were about 20- to 110-fold higher at 24 hpi, and 25- to 1400-fold higher at 36 hpi; in KO-IFNAR1 cells infected with DTMUV, the induction of these five genes was much suppressed at the same time points, with about 5- to 25-fold induction at 24 hpi, and 5- to 150-fold induction at 36 hpi (Figure 2A). In IBV-infected wild type DF-1 cells, the induction levels of these five ISGs were about 60-fold to more than 500-fold at 24 hpi, and about 25-fold to more than 420-fold at 36 hpi; in IBV-infected KO-IFNAR1 cells, their expression levels were about 3-fold to more than 190-fold at 24 hpi, and about 5-fold to more than 100-fold at 36 hpi (Figure 2B). Similarly, in NDV-infected DF-1 cells, the induction levels of IFN-β and four ISGs were about 40- to 90-fold higher at 24 hpi, and 15- to 102-fold higher at 36 hpi; in NDV-infected KO-IFNAR1 cells, their induction levels at 24 hpi were about 5-fold to over 38-fold, and at 36 hpi were about 8-fold to over 35-fold (Figure 2C). After overexpression of chIFNAR1 in KO-IFNAR1 cells, the RNA levels of ISGs were almost restored to similar levels to those in the DF-1 group. In contrast, in DF-1 cells treated with UV-inactivated virus, the expression of ISGs was almost undetectable. Taken together, these results demonstrate that knockout of chIFNAR1 significantly suppressed the induction of type I IFNs and related ISGs, confirming the functional impairment of the type I IFN pathway in the knockout cells.

### 3.3. Knockout of chIFNAR1 Promotes the Replication of DTMUV, IBV and NDV

**Figure 3:**
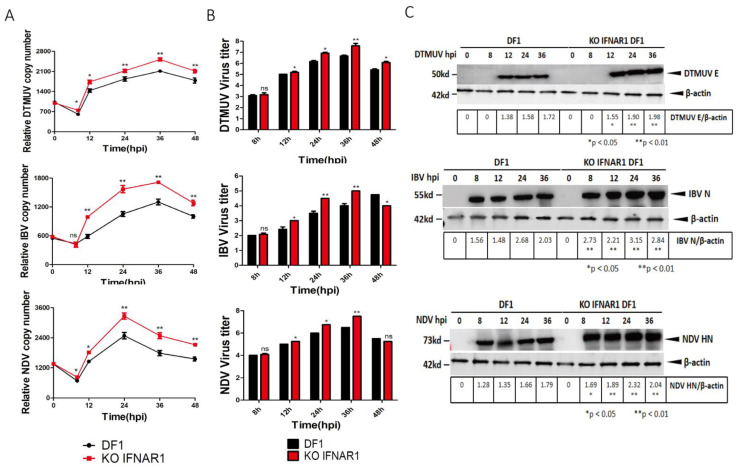
Knockout of KO-IFNAR1 promotes the replication of DTMUV, IBV and NDV. (**A**) Wild type (DF-1) and IFNAR1-knockout (KO-IFNAR1) cells were infected with DTMUV, IBV and NDV at an MOI~ 1, and harvested at 0, 8, 12, 24, 36 and 48 hpi, respectively. Genomic RNA levels of DTMUV, IBV and NDV were determined by RT-qPCR and presented. * *p* < 0.05; ** *p* < 0.01; ns, non-significant. (**B**) Viral titers were determined by TCID50(Log10TCID50/0.1mL) from the extracellular fluids of DF-1 and KO-IFNAR1 cells harvested as in (**A**). Error bar represents the standard error of three replicate experiments. T tests were conducted between DF-1 (black) and KO-IFNAR1 (red) cells. * *p* < 0.05; ** *p* < 0.01; ns, non-significant. (**C**) Total cell lysates were prepared in parallel. The replication of DTMUV, IBV and NDV were assessed by Western blot with anti-DTMUV-E, anti-IBV-N and anti-NDV-HN antibodies. β-actin was detected as a loading control. * *p* < 0.05; ** *p* < 0.01; ns, non-significant.

The effect of chIFNAR1-knockout on viral replication was then assessed by infection of wild type DF-1 and KO-IFNAR1 cells with DTMUV, IBV and NDV, respectively. Cells were harvested at indicated time points, levels of viral RNA and viral proteins were determined by RT-qPCR and Western blot, and viral titers in the culture media were determined by TCID50. The RT-qPCR results confirmed that, by comparing with those in DF-1 cells (black), DTMUV RNA levels in KO-IFNAR1 cells (red) were reduced 20- to 30-fold at 12 and 48 hpi, IBV RNA levels in KO IFNAR1 cells (red) were reduced 20- to 60-fold at 12 and 48 hpi, and NDV RNA levels in KO IFNAR1 cells (red) were reduced 30- to 60-fold at 12 and 48 hpi (Figure 3A). The viral protein levels were consistent with the viral RNA levels (Figure 3B), and virus titers in media collected from DTMUV-, IBV- and NDV-infected KO IFNAR1 (red) were 10- to 30-fold, 15- to 50-fold and 6- to 10- fold higher than those in DF-1 cells at 24 and 36 hpi, respectively (Figure 3C). In addition, overexpression of IFNAR1 in KO IFNAR1 cells reduced the replication of DTMUV, IBV and NDV to a level similar to that in DF-1 cells (Figure S5). These results demonstrate that knockout of IFNAR1 significantly enhances the replication of these three common poultry viruses.

### 3.4. Transcriptomic Analysis of Differential Gene Expression in DF-1 and KO-IFNAR1 Cells Infected with DTMUV

DTMUV-infected DF-1 and KO-IFNAR1 cells were harvested at 24 hpi, total RNA extracted and subjected to transcriptomic analysis. Cells treated with UV-DTMUV were included as mock controls. Host gene expression in infected and control cells was normalized to the internal GAPDH transcript, and the ratio of each transcript was calculated at each time point and presented at a *p*-value < 0.05 and a threshold of |log2 (fold change)| > 1.

**Figure 4:**
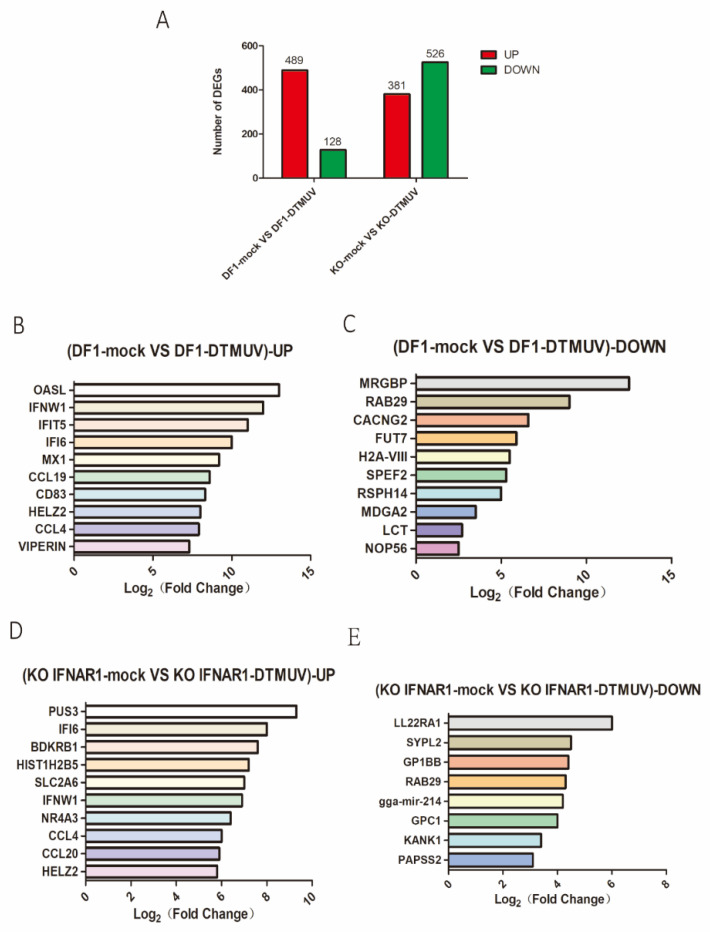
Transcriptomic analysis of differential gene expression in DF-1 and KO-IFNAR1 cells infected with DTMUV. (**A**) The number of up-regulated genes in DTMUV-infected cells at 24 hpi are shown in red, and the down-regulated genes in green. (**B**) Top 10 genes with the highest expression levels among all up-regulated genes in the DF-1 infection group transcriptome data. (**C**) The 10 genes with lowest expression levels among all down-regulated genes in DF-1 infection group transcriptome data. (**D**) Top 10 genes with the highest expression level among all up-regulated genes in the KO-IFNAR1 infection group transcriptome data. (**E**) The 10 genes with lowest expression levels among all down-regulated genes in KO-IFNAR1-infected transcriptome data.

In DTMUV-infected DF-1 cells, 617 DEGs, including 489 up-regulated and 128 down-regulated, were identified in the infection group (Figure 4A). In DTMUV-infected KO-IFNAR1 cells, 907 DEGs were identified, with 381 up-regulated and 526 down-regulated (Figure 4A).

Among all up-regulated genes in the total transcriptome data, the 10 most up-regulated genes in the DF-1 group were OASL, IFNW1, IFIT5, IFI6, MX1, CCL19, CD83, HELZ2, CCL4 and VIPERIN (Figure 4B), and the 10 most significantly down-regulated genes were MRGBP, RAB29, CACNG2, FUT7, H2A-VIII, SPEF2, RSPH14, MDGA2, LCT and NOP56 (Figure 4C). The 10 most up-regulated genes in the KO-IFNAR1 group were PUS3, IFI6, BDKRB1, HIST1H2B5, SLC2A6, IFNW1, NR4A3, CCL4, CCL20 and HELZ2 (Figure 4D), and the eight most significantly down-regulated genes were LL22RA1, SYPL2, GP1BB, RAB29, gga-mir-214, GPC1, KANK1 and PAPSS2 (Figure 4E).

### 3.5. Biological Significance of DEGs in DTMUV-Infected KO-IFNAR1 Cells

**Figure 5:**
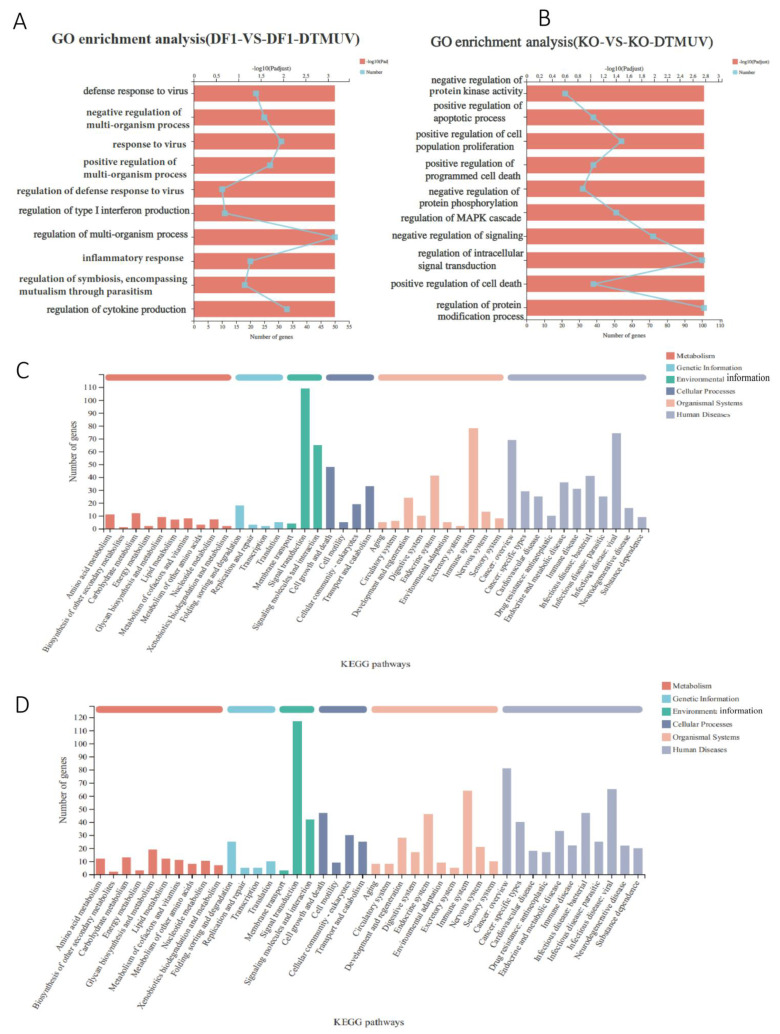
GO enrichment analysis and KEGG analysis of differentially expressed genes in DTMUV—infected DF-1 and KO-IFNAR1 cells. Genes in DF-1 (**A**) and KO-IFNAR1 (**B**) cells induced by DTMUV infection were classified using the GO system. Genes induced by DTMUV infection in DF-1 (**C**) and KO-IFNAR1 (**D**) cells were classified using the KEGG system.

Classification of DEGs by the Gene Ontology (GO) system showed that the involved molecular functions and biological processes included defense response to viruses, regulation of IFN-I production, regulation of multi-organism processes, inflammatory response and regulation of cytokine production in the DTMUV-infected DF-1 group (Figure 5A). In the DTMUV-infected KO-IFNAR1 group, the involved molecular functions and biological processes included negative regulation of protein kinase activity, positive regulation of apoptotic process, positive regulation of cell population proliferation, negative regulation of protein phosphorylation and regulation of intracellular signal transduction (Figure 5B). These results demonstrated that in DTMUV-infected DF-1 cells, most DEGs were functionally involved in the defense response to virus infection and the immune system, while most genes in KO-IFNAR1 cells were involved in cell proliferation and apoptosis. It suggests that cells may rely on other compensatory mechanisms to resist virus invasion upon knockout of the IFNAR1 gene.

KEGG pathway annotation of the screened DEGs was carried out to determine important metabolic and signal transduction pathways involved. DTMUV infection of DF-1 and KO-IFNAR1 cells activated pathways related to cell growth and death, immune system, infectious diseases: virus, and signal transduction (Figure 5C,D). In the cell growth and death pathway, NGF, TNSRSF 10B, MYC, ZFP36L1, NFKBIA, SESN2, JUN and FOS were significantly up-regulated in the KO-IFNAR1 group, while in the DF-1 group, TLR3, IFNK, IFNW1, IL6, IL1B and STAT2 were significantly up-regulated (Figure S4A). In the immune system pathway, PLK2, JUN and FOS were significantly up-regulated in the KO-IFNAR1 group, while IRF7, IRF1, IL6, TRIM25, DHX58 and CCL19 were significantly up-regulated in the DF-1 group (Figure S4B). In the signal transduction pathway, MYC, JUN, FOS, DUSP1 and EGR1 were significantly up-regulated in the KO-IFNAR1 group, while IRF1, IFNW1, CCL20, IL6 and TRIM25 were significantly up-regulated in the DF-1 group (Figure S4C). In infectious diseases: virus pathway, MYC, JUN, FOS, HSPA2, NFKBIA, CCL4 and EGR1 were significantly up-regulated in the KO IFNAR1 group, while in the DF-1 group, IRF7, IRF1, MX1, TRIM25, RSAD2, IL6, OASL and IFNW1 were significantly up-regulated (Figure S4D). It was noted that in all four pathways, MYC, JUN, FOS and EGR1 were identified in DTMUV-infected KO-IFNAR1 cells. As these genes are mainly involved in the regulation of cell growth and apoptosis, it suggests that when the IFN-I function is impaired, the main cellular response to DTMUV infection is to regulate apoptosis, which may, in turn, regulate viral replication.

### 3.6. Validation of Transcriptomic Data by RT-qPCR

**Figure 6:**
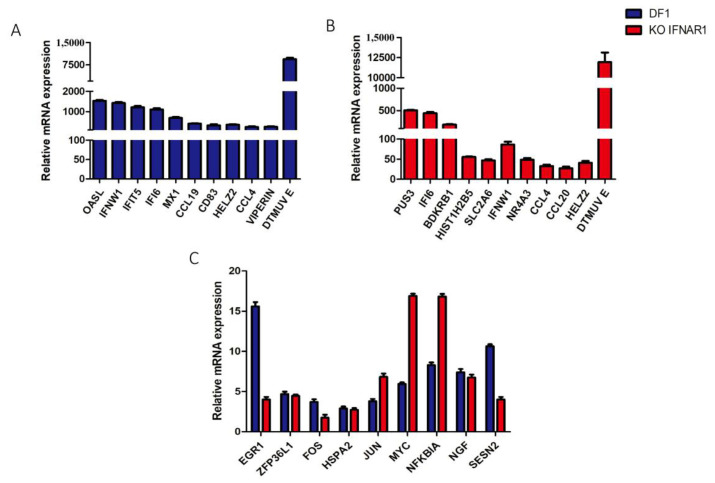
Validation of up-regulated genes in DTMUV-infected DF-1 and KO-IFNAR1 cells by RT-qPCR. (**A**) Fold-induction of the top 10 significantly up-regulated genes in DF-1 cells infected with DTMUV. (**B**) Fold-induction of the top 10 significantly up-regulated genes in KO-IFNAR1 cells infected with DTMUV. (**C**) Fold-induction of EGR1, ZFP36L1, FOS, HSPA2, JUN, MYC, NFKBIA, NGF and SESN2 in DF-1 and KO-IFNAR1 cells infected with DTMUV.

To validate the transcriptomic data, DF-1 and KO-IFNAR1 cells were infected with DTMUV and mock-treated with UV-DTMUV, respectively, harvested at the indicated time points, and followed by RNA extraction and RT-qPCR analysis. The results showed that RNA levels of the top 10 most significantly up-regulated genes by DTMUV-infected DF-1 (Figure 6A) and KO-IFNAR1 cells (Figure 6B) were consistent with the transcriptomic results.

As EGR1, ZFP36L1, FOS, HSPA2, JUN, MYC, NFKBIA, NGF and SESN2 were up-regulated in both DTMUV-infected DF-1 and KO-IFNAR1 cells, the up-regulation of these genes was also verified by qPCR. The results showed that JUN, MYC and NFKBIA were up-regulated by 6-, 11- and 9-fold, respectively, in DTMUV-infected KO-IFNAR1, compared with those in DF-1 cells (Figure 6C,D). However, 11- and 6-fold less induction of EGR1 and SESN2, respectively, were observed in DTMUV-infected KO-IFNAR1, compared with those in DF-1 cells (Figure 6C,D). These results suggest that MYC, JUN and NFKBIA may be the main genes induced by DTMUV infection when the IFN-I function is inhibited, which may regulate apoptosis and viral replication.

### 3.7. Knockdown of HELZ2 and IFI6 Promotes DTMUV Replication

**Figure 7:**
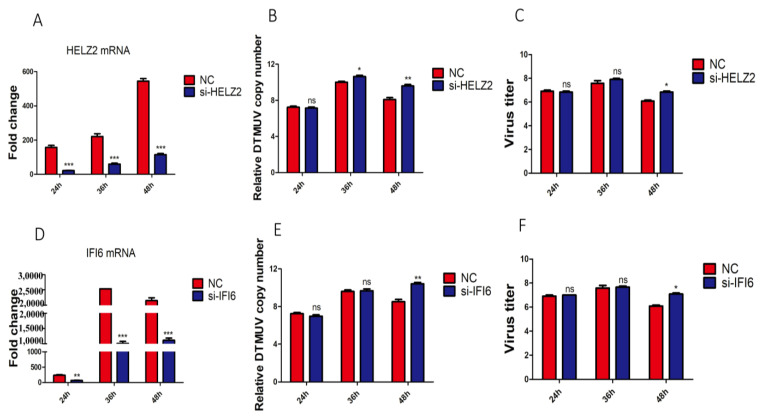
Effects of knockdown of HELZ2 and IFI6 on DTMUV replication in KO-IFNAR1 cells. (**A**) Relative mRNA levels of HELZ2 in HELZ2-knockdown KO-IFNAR1 cells infected with DTMUV. Cells were transfected with siHELZ2 and siNC for 5 h, respectively, and infected with DTMUV at an MOI ~1, harvested at indicated time points for RT-qPCR with specific primers to determine relative mRNA levels. (**B**) Relative viral RNA levels(Normalized DTMUV copy number (log10) per μg of total RNA) harvested from HELZ2-knockdown (siHELZ2) or negative control (siNC) cells at indicated times after DTMUV infection. (**C**) Viral titers were determined by TCID50 from the extracellular fluids of cells harvested as in (**B**). Error bar represents the standard error of three replicate experiments. (**D**) Relative mRNA levels of IFI6 in IFI6-knockdown KO-IFNAR1 cells infected with DTMUV. Cells were transfected with siIFI6 and siNC for 5 h, respectively, and infected with DTMUV at an MOI ~1, harvested at indicated time points for RT-qPCR with specific primers to determine relative mRNA levels. (**E**) Relative viral RNA levels(Normalized DTMUV copy number (log10) per μg of total RNA) harvested from IFI6-knockdown (siIFI6) or negative control (siNC) cells at indicated times after DTMUV infection. (**F**) Viral titers were determined by TCID50 from the extracellular fluids of cells harvested as in (**E**). Error bar represents the standard error of three replicate experiments. * *p* < 0.05; ** *p* < 0.01, *** *p* < 0.001; ns, non-significant.

Since HELZ2 and IFI6 were significantly up-regulated by DTMUV infection of both DF-1 and KO-IFNAR1 cells, the antiviral functions of these two genes were studied by transient knockdown in KO-IFNAR1 cells with siRNA, and the effects of HELZ2- and IFI6-knockdown on DTMUV replication were analyzed by RT-qPCR. Efficient knockdown of HELZ2 in KO-IFNAR1 cells resulted in a 4- to 60-fold increase in viral RNA levels and viral titers at 36 and 48 hpi, respectively (Figure 7A–C), and efficient knockdown led to a 100-fold increase in viral RNA levels and viral titers at 48 hpi (Figure 7C–F). These results demonstrate that HELZ2 and IFI6 may be involved in regulating the replication of DTMUV at late infection stages in cells when IFN-I functions are compromised.

## 4. Discussion

In this study, we show that IFNAR1-knockout DF-1 cell line supports much more efficient replication of DTMUV, NDV and IBV. IFN exerts its intracellular activity by binding to IFNRs on the cell surface, initiating a series of downstream immune cascades and resulting in the efficient expression of ISGs. Knockout of IFNAR1 gene in DF-1 cells significantly inhibited the expression of many DTMUV-induced ISGs, including VIPERIN, IFIT5, OASL and MX, which may be responsible for the increased replication of DTMUV, NDV and IBV in the KO-IFNAR1 cell line. This is consistent with previous studies that the IFN-I-mediated expression of OASL, VIPERIN, IFIT5 and MX inhibits the replication of DTMUV [4,16], and knockdown of chicken OASL increases NDV titers in DF-1cells [17].

Transcriptomic analysis demonstrated that DTMUV infection of KO-IFNAR1 cells primarily activates apoptosis-related pathways, such as MAPK. Several downstream factors of the MAPK signaling pathway, such as c-JUN, c-FOS, MYC and EGR1, were significantly up-regulated, pointing to a potential antiviral role of this pathway and its downstream proteins. In fact, it was reported that signal molecules of the MAPK pathway, including AP-1, c-FOS, JUN, JunD, MAX, and c-Myc, were significantly up-regulated after the highly pathogenic avian influenza virus (HPAIV)-resistant chicken lines were infected with AIV [7], suggesting that the antiviral activity of HPAIV-resistant chicken lines was mainly mediated by the MAPK signaling pathway. Zhang et al. showed that knockdown of the p53 gene can up-regulate the expression of c-MYC and inhibit apoptosis, thereby favoring the replication of avian leukosis virus subgroup J (ALV-J) [18]. Infectious spleen and kidney necrosis virus (ISKNV) and rhabdovirus (SCRV) activate the p53/c-MYC pathway, but the up-regulation of c-MYC appears to promote viral replication [19].

c-JUN and c-FOS are major components of the dimeric transcription factor AP-1. Zhu et al. suggested that overexpression of c-JUN or its upstream kinase could increase IBV-induced mRNA expression of the proinflammatory factor IL-8 [20]. Influenza A virus (IAV) infection activates the AP-1 signaling pathway, promotes pro-IL-1β mRNA transcription and activates the NLRP3 inflammasome, a process associated with antiviral host defense and inflammatory diseases [21]. EGR1 can up-regulate the expression of the IFN-regulated antiviral (IRAV) promoter to inhibit porcine epidemic diarrhea virus (PEDV) replication [22]. Conversely, EGR1 is up-regulated during bovine herpesvirus 1 (BHV-1) infection, and overexpression of EGR1 promotes BHV-1 replication [23].

In this study, HELZ2 and IFI6 are found to be strongly induced in both DTMUV-infected DF-1 and KO-IFNAR1 cells, suggesting that these two genes can be induced by both IFN-I-dependent and -independent pathways. HELZ2 can interact with the nuclear receptor Aryl Hydrocarbon Receptor (AHR) and regulate immune responses and lipid metabolism, thereby reducing dengue virus (DENV) infectivity [24]. Knockdown of HELZ2 in KO-IFNAR1 could further increase the DTMUV RNA level at later stages of infection. However, HELZ2 cannot restrict Zika virus (ZIKV) propagation in osteoblast-like Saos-2 cells [25] and AIV infection could not induce the expression of HELZ2 in DF-1cells [26]. IFI6 inhibits the replication of multiple flaviviruses, including Yellow Fever Virus (YFV), West Nile Virus (WNV), DENV and ZIKV [27]. Consistent with our results, IFI6 does not affect the early transcription and translation, but could delay RNA replication and reduce transcription and translation of viral genes at late stages [28]. IFI6 overexpression prevents YFV-induced ER invagination, acting indirectly by preventing flaviviruses from establishing favorable conditions for replication [29].

At present, the application of CRISPR/Cas9 in avian species is only in its infancy. Previous studies have shown that the mutation efficiency in chicken cells is generally low [30,31]. This study showed that both sgRNAs could be used to effectively edit the IFNAR1 gene in DF-1 cells, but the one closer to the 5’ end would be more likely introducing frameshifting deletion, which would lead to premature termination of protein translation [9], so the sgRNA targeting the second exon at the 5’ end of the gene was selected. Meanwhile, we have assessed the off-target rate of the sgRNA (ACCTAATGTGGAACTACACTGG) on the website (http://crispor.tefor.net/crispor.py?batchId=tt8oz1UDbnIe30fclvaR)(accessed on 22 February 2022) and showed the location of the most likely off-target sites. In addition, our results show that overexpression of IFNAR1 (68KD) in KO IFNAR1 cells can effectively inhibit virus replication, but the function of IFNAR1 may not be fully restored due to the transfection efficiency of chicken-derived cells and the insertion of the flag tag.

In conclusion, knockout of IFNAR1 efficiently enhances the replication of DTMUV, NDV and IBV. DTMUV infection of the knockout cells activates apoptosis-related pathways, such as the MAPK pathway. This study provides a tool for further research on the molecular mechanism of antiviral innate immune response in poultry, and a new potential cell resource for efficient production of cell-derived vaccines against avian viruses.

## Figures and Tables

**Table 1:** The gene specific primers for qPCR.

	Gene	Foward Primer (5′-3′)	Reverse Primer (5′-3′)
Primers for qPCR	GAPDH (C)	GCCATCACAGCCACACAGA	TTTCCCCACAG CCTTAGCA
	VIPERIN (C)	TCGTTCTGCCTCTGCTCTCCTG	TTGTAGTTGCACTGCCTGGTGAAG
	IFIT5 (C)	CACCAGCTAGGACTCTGCTACCG	CCTCCGCATACATC CTTGCCAAG
	MX (C)	CTGCGGACAAGCCATAGAA	GCACCCCAAAAACTCCTACA
	OASL (C)	ATCATCGAGAG GCGGCTC	TCCGCGCTACAAGGACAGC
	IFN-β (C)	AGATGGCTCCCAGCTCTACA	AGTGGTTGAGCTGGTTG AGG
	NDV-NP	GGAAGGAAGCGGAGCCATCATG	GCTGTGGAGGGTTCATCTCATTCG
	DTMUV-E	CGCTGAGATGGAGGATTATGG	ACTGATTTTTGGTGG CGTG
	IBV-N	GTTCTCGCATAAGGTCGGCTA	GCTCACTAAACACCACCAGAAC

## Data Availability

The original contributions presented in the study are publicly available. These data can be found here: https://www.ncbi.nlm.nih.gov/sra/PRJNA825282 (accessed on 11 June 2022) and https://www.ncbi.nlm.nih.gov/sra/PRJNA858867 (accessed on 14 July 2022).

## Supplementary Materials

The following supporting information can be downloaded at: https://www.mdpi.com/article/10.3390/v14102225/s1, Figure S1: Construction of pX459 gene knockout plasmid; Figure S2: Validation of sgRNA knockout efficiency; Figure S3: 2-base deletion results in premature termination of IFNAR1 protein translation; Figure S4: Heatmap analysis of differentially expressed genes; Figure S5: Overexpression of IFNAR1 restores IFNAR1 function in KO IFNAR1 cells.

## References

[1] Randall R.E., Goodbourn S. (2008). Interferons and viruses: An interplay between induction, signalling, antiviral responses and virus countermeasures. J. Gen. Virol..

[2] Ahmad S., Alsayed Y.M., Druker B.J., Platanias L.C. (1997). The type I interferon receptor mediates tyrosine phosphorylation of the CrkL adaptor protein. J. Biol. Chem..

[3] Stark G.R., Darnell J.E. (2012). The JAK-STAT Pathway at Twenty. Immunity.

[4] Xiang C., Huang M., Xiong T., Rong F., Li L., Liu D.X., Chen R.A. (2020). Transcriptomic Analysis and Functional Characterization Reveal the Duck Interferon Regulatory Factor 1 as an Important Restriction Factor in the Replication of Tembusu Virus. Front. Microbiol..

[5] Fung T.S., Liu D.X. (2017). Activation of the c-Jun NH2-terminal kinase pathway by coronavirus infectious bronchitis virus promotes apoptosis independently of c-Jun. Cell Death Dis..

[6] Yuan L.X., Liang J.Q., Zhu Q.C., Dai G., Li S., Fung T.S., Liu D.X. (2021). A gammacoronavirus, avian infectious bronchitis virus, and an alphacoronavirus, porcine epidemic diarrhea virus, exploit a cell survival strategy by upregulating cFOS to promote virus replication. J. Virol..

[7] Vu T.H., Hong Y., Truong A.D., Lee S., Heo J., Lillehoj H.S., Hong Y.H. (2022). The highly pathogenic H5N1 avian influenza virus induces the MAPK signaling pathway in the trachea of two Ri chicken lines. Anim. Biosci..

[8] Cong L., Ran F.A., Cox D., Lin S., Barretto R., Habib N., Hsu P.D., Wu X., Jiang W., Marraffini L.A. (2013). Multiplex Genome Engineering Using CRISPR/Cas Systems. Science.

[9] Antonova E., Glazova O., Gaponova A., Eremyan A., Zvereva S., Grebenkina N., Volkova N., Volchkov P. (2018). Successful CRISPR/Cas9 mediated homologous recombination in a chicken cell line. F1000Research.

[10] Hsu P.D., Lander E.S., Zhang F. (2014). Development and Applications of CRISPR-Cas9 for Genome Engineering. Cell.

[11] Li B., Dewey C.N. (2011). RSEM: Accurate transcript quantification from RNA-Seq data with or without a reference genome. BMC Bioinform..

[12] Ti J., Zhang L., Li Z., Zhao D., Zhang Y., Li F., Diao Y. (2015). Effect of age and inoculation route on the infection of duck Tembusu virus in Goslings. Vet. Microbiol..

[13] Xie C., Mao X., Huang J., Ding Y., Wu J., Dong S., Kong L., Gao G., Li C.Y., Wei L. (2011). KOBAS 2.0: A web server for annotation and identification of enriched pathways and diseases. Nucleic Acids Res..

[14] Langfelder P., Horvath S. (2008). WGCNA: An R package for weighted correlation network analysis. BMC Bioinform..

[15] Shannon P., Markiel A., Ozier O., Baliga N.S., Wang J.T., Ramage DAmin N., Schwikowski B., Ideker T. (2003). Cytoscape: A software environment for integrated models of biomolecular interaction networks. Genome Res..

[16] Chen S., Zhang W., Wu Z., Zhang J., Wang M., Jia R., Zhu D., Liu M., Sun K., Yang Q. (2017). Goose Mx and OASL Play Vital Roles in the Antiviral Effects of Type I, II, and III Interferon against Newly Emerging Avian Flavivirus. Front. Immunol..

[17] Del V.A., Jang H.J., Monson M.S., Lamont S.J. (2021). Role of the chicken oligoadenylate synthase-like gene during in vitro Newcastle disease virus infection. Poult. Sci..

[18] Zhang H., Zhang H., Cao S., Sui C., Song Y., Zhao Y., Liu S. (2021). Knockout of p53 leads to a significant increase in ALV-J replication. Poult. Sci..

[19] Ye C., Liu S., Li N., Zuo S., Niu Y., Lin Q., Liang H., Luo X., Fu X. (2022). Mandarin Fish (Siniperca chuatsi) p53 Regulates Glutaminolysis Induced by Virus via the p53/miR145-5p/c-Myc Pathway in Chinese Perch Brain Cells. Microbiol. Spectr..

[20] Zhu Q.C., Li S., Yuan L.X., Chen R.A., Liu D.X., Fung T.S. (2021). Induction of the Proinflammatory Chemokine Interleukin-8 Is Regulated by Integrated Stress Response and AP-1 Family Proteins Activated during Coronavirus Infection. Int. J. Mol. Sci..

[21] Wan P., Zhang S., Ruan Z., Liu X., Yang G., Jia Y., Li Y., Pan P., Li W., Li G. (2022). AP-1 signaling pathway promotes pro-IL-1β transcription to facilitate NLRP3 inflammasome activation upon influenza A virus infection. Virulence.

[22] Wang H., Kong N., Jiao Y., Dong S., Sun D., Chen X., Zheng H., Tong W., Yu H., Yu L. (2021). EGR1 Suppresses Porcine Epidemic Diarrhea Virus Replication by Regulating IRAV To Degrade Viral Nucleocapsid Protein. J. Virol..

[23] Hou P., Zhao M., He W., He H., Wang H. (2019). Cellular microRNA bta-miR-2361 inhibits bovine herpesvirus 1 replication by directly targeting EGR1 gene. Vet. Microbiol..

[24] Fusco D.N., Pratt H., Kandilas S., Cheon S.S.Y., Lin W., Cronkite D.A., Basavappa M., Jeffrey K.L., Anselmo A., Sadreyev R. (2017). HELZ2 Is an IFN Effector Mediating Suppression of Dengue Virus. Front. Microbiol..

[25] Drouin A., Wallbillich N., Theberge M., Liu S., Katz J., Bellovodad K., Cheon S.S.Y., Gootkinde F., Bierman E., Zavrase J. (2021). Impact of Zika virus on the human type I interferon osteoimmune response. Cytokine.

[26] Yu S., Mao H., Jin M., Lin X. (2020). Transcriptomic Analysis of the Chicken MDA5 Response Genes. Genes.

[27] Berthoux L. (2020). The Restrictome of Flaviviruses. Virol. Sin..

[28] Richardson R.B., Ohlson M.B., Eitson J.L., Kumar A., McDougal M.B., Boys I.N., Mar K.B., de la Cruz-Rivera P.C., Douglas C., Konopka G. (2018). A CRISPR screen identifies IFI6 as an ER-resident interferon effector that blocks flavivirus replication. Nat. Microbiol..

[29] Arakawa M., Morita E. (2019). Flavivirus Replication Organelle Biogenesis in the Endoplasmic Reticulum: Comparison with Other Single-Stranded Positive-Sense RNA Viruses. Int. J. Mol. Sci..

[30] Zhang Y., Wang Y., Zuo Q., Li D., Zhang W., Wang F., Ji Y., Jin J., Lu Z., Wang M. (2017). CRISPR/Cas9 mediated chicken Stra8 gene knockout and inhibition of male germ cell differentiation. PLoS ONE.

[31] Zuo Q., Wang Y., Cheng S., Lian C., Tang B., Wang F., Lu Z., Ji Y., Zhao R., Zhang W. (2016). Site-Directed Genome Knockout in Chicken Cell Line and Embryos Can Use CRISPR/Cas Gene Editing Technology. G3.

