# Supplementary material for: Construction and Transcriptomic Study of Chicken IFNAR1-Knockout Cell Line Reveals the Essential Roles of Cell Growth- and Apoptosis-Related Pathways in Duck Tembusu Virus Infection
Source: Viruses. 2022 Oct 9;14(10):2225. doi: 10.3390/v14102225 (PMC9611459; doi:10.3390/v14102225)
Supplement: Supplementary file 1 [file viruses-14-02225-s001.zip › supplementary/WB original.pdf]

FIGURE 3C

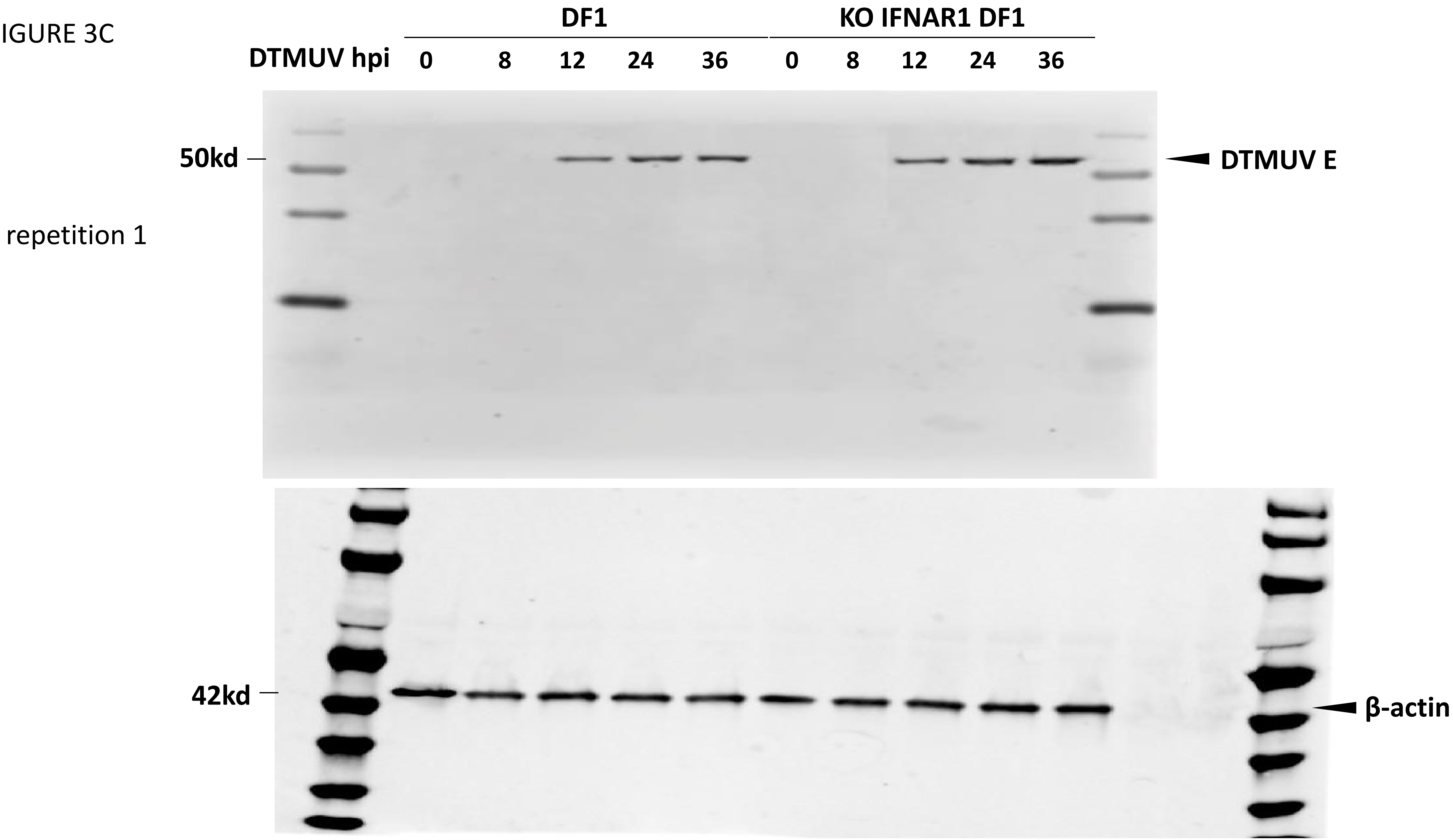

DF1
KO IFNAR1 DF1
DTMUV hpi
0
8
12
24
36
0
8
12
24
36
50kd
DTMUV E
repetition 1
42kd
β-actin

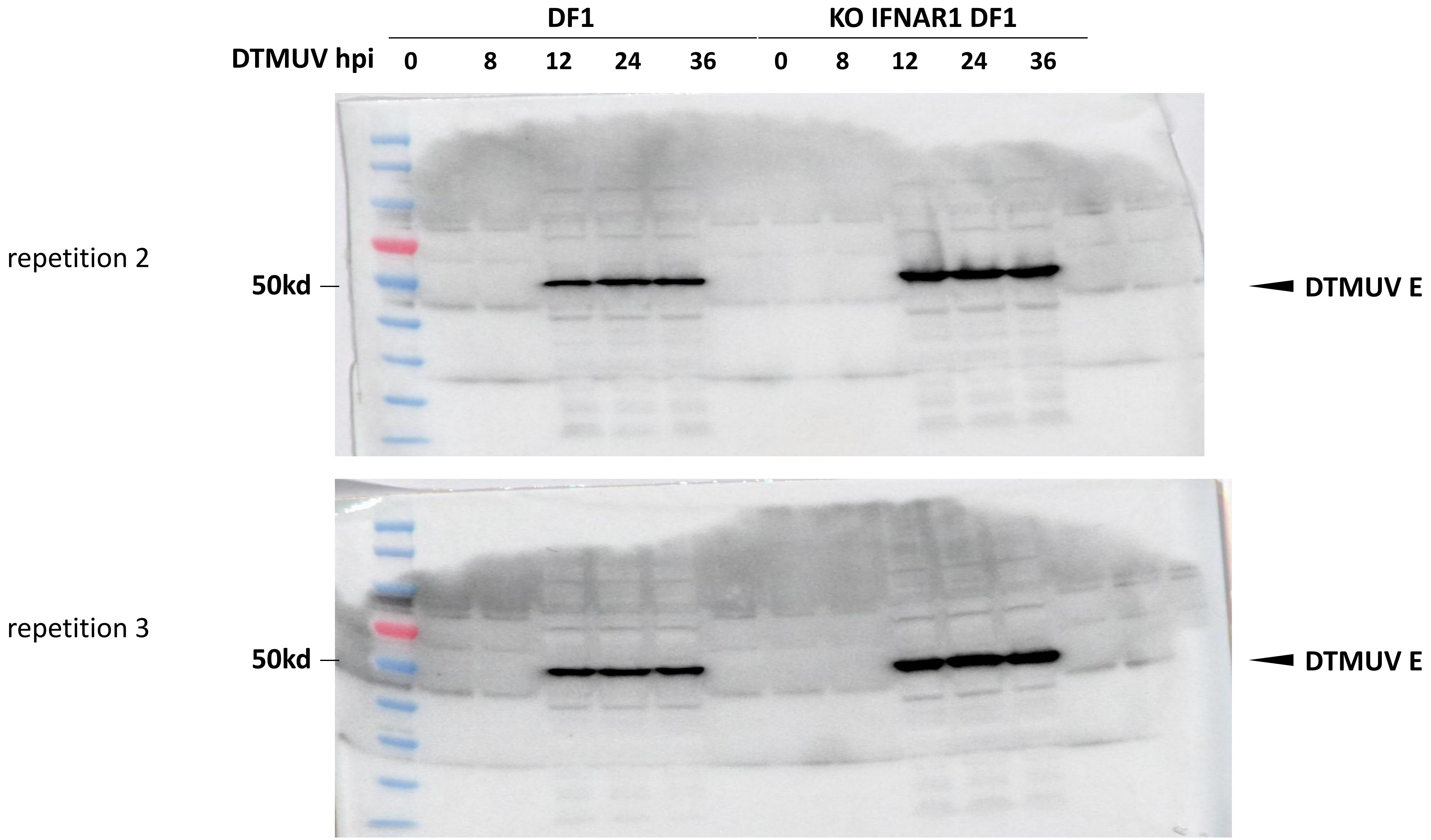

DF1
KO IFNAR1 DF1
DTMUV hpi
0
8
12
24
36
0
8
12
24
36
repetition 2
50kd
DTMUV E
repetition 3
50kd
DTMUV E

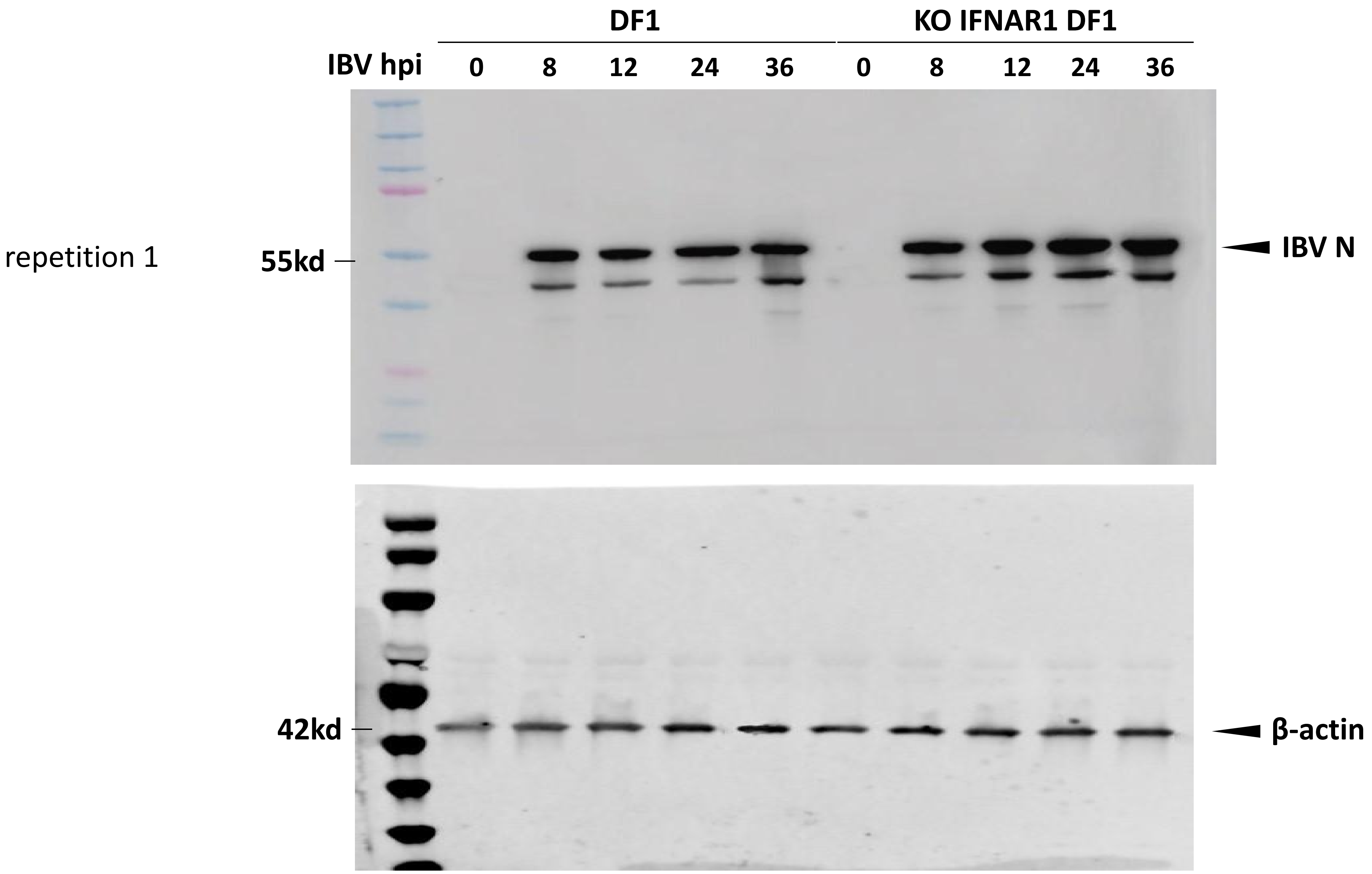

DF1
KO IFNAR1 DF1
IBV hpi
0
8
12
24
36
0
8
12
24
36
repetition 1
55kd
IBV N
42kd
β-actin

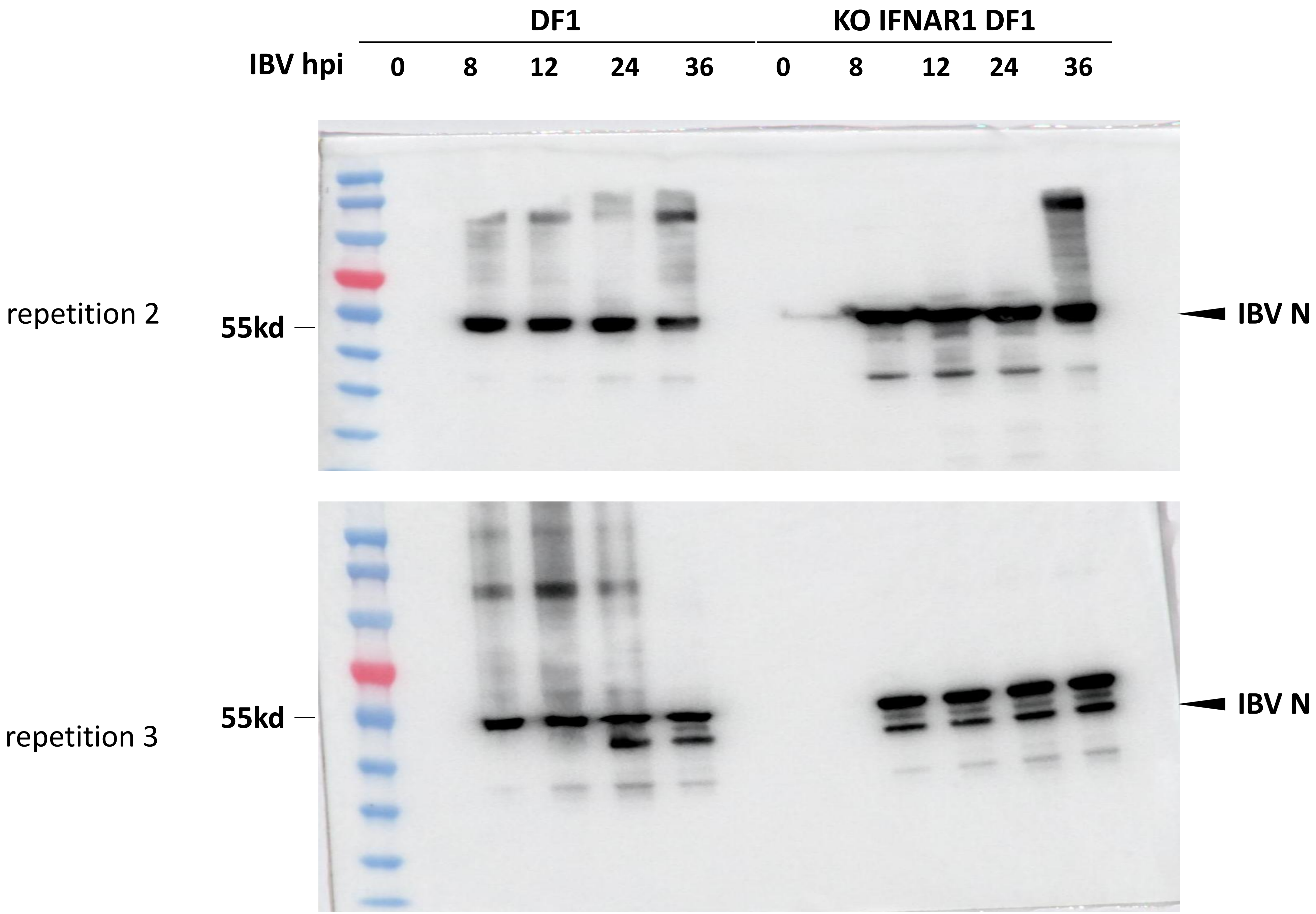
DF1
KO IFNAR1 DF1
IBV hpi
0
8
12
24
36
0
8
12
24
36
repetition 2
55kd
IBV N
repetition 3
55kd
IBV N

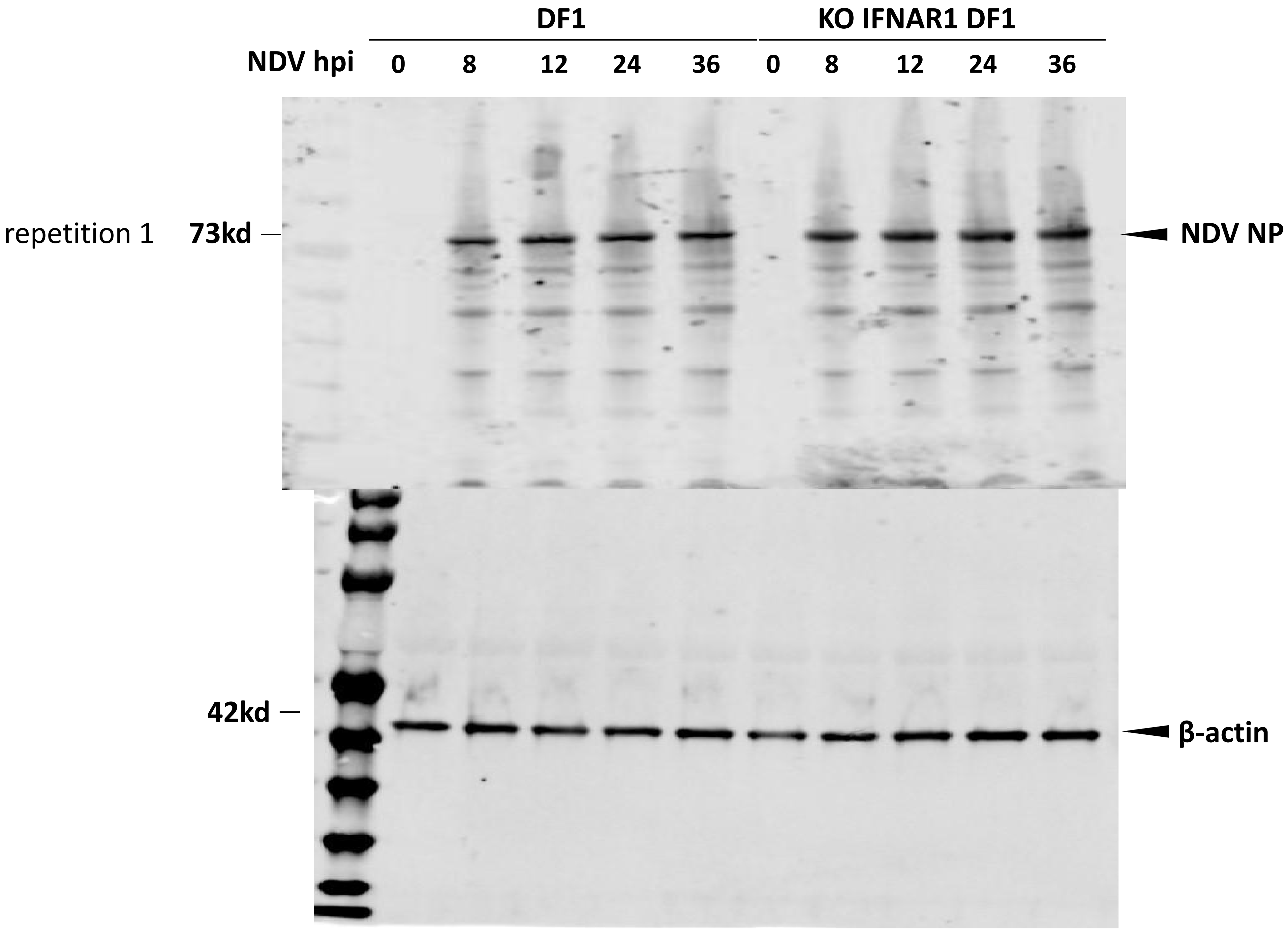
DF1
KO IFNAR1 DF1
NDV hpi
0
8
12
24
36
0
8
12
24
36
repetition 1
73kd
NDV NP
42kd
β-actin

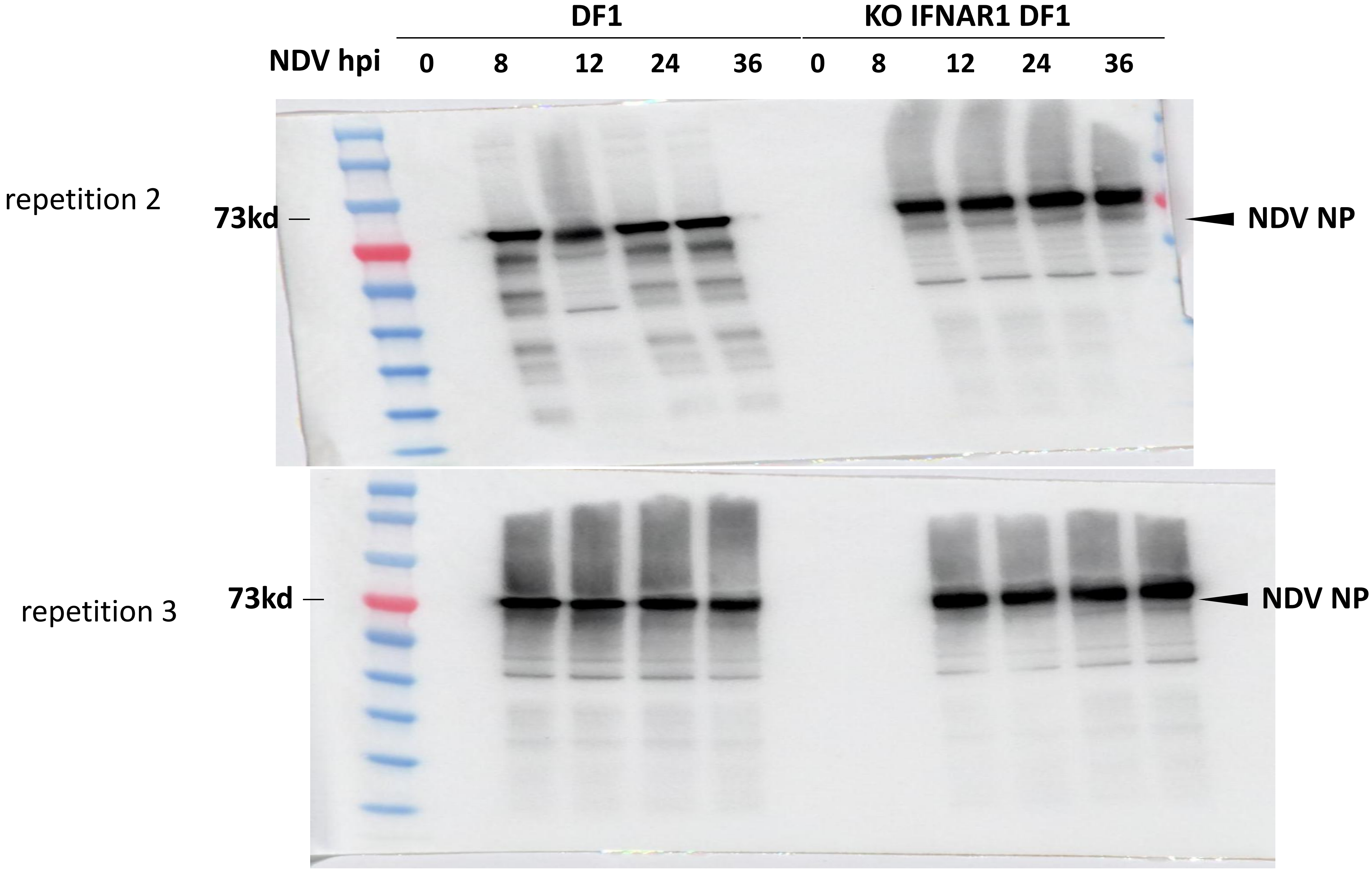
DF1
KO IFNAR1 DF1
NDV hpi
0
8
12
24
36
0
8
12
24
36
repetition 2
73kd
NDV NP
repetition 3
73kd
NDV NP

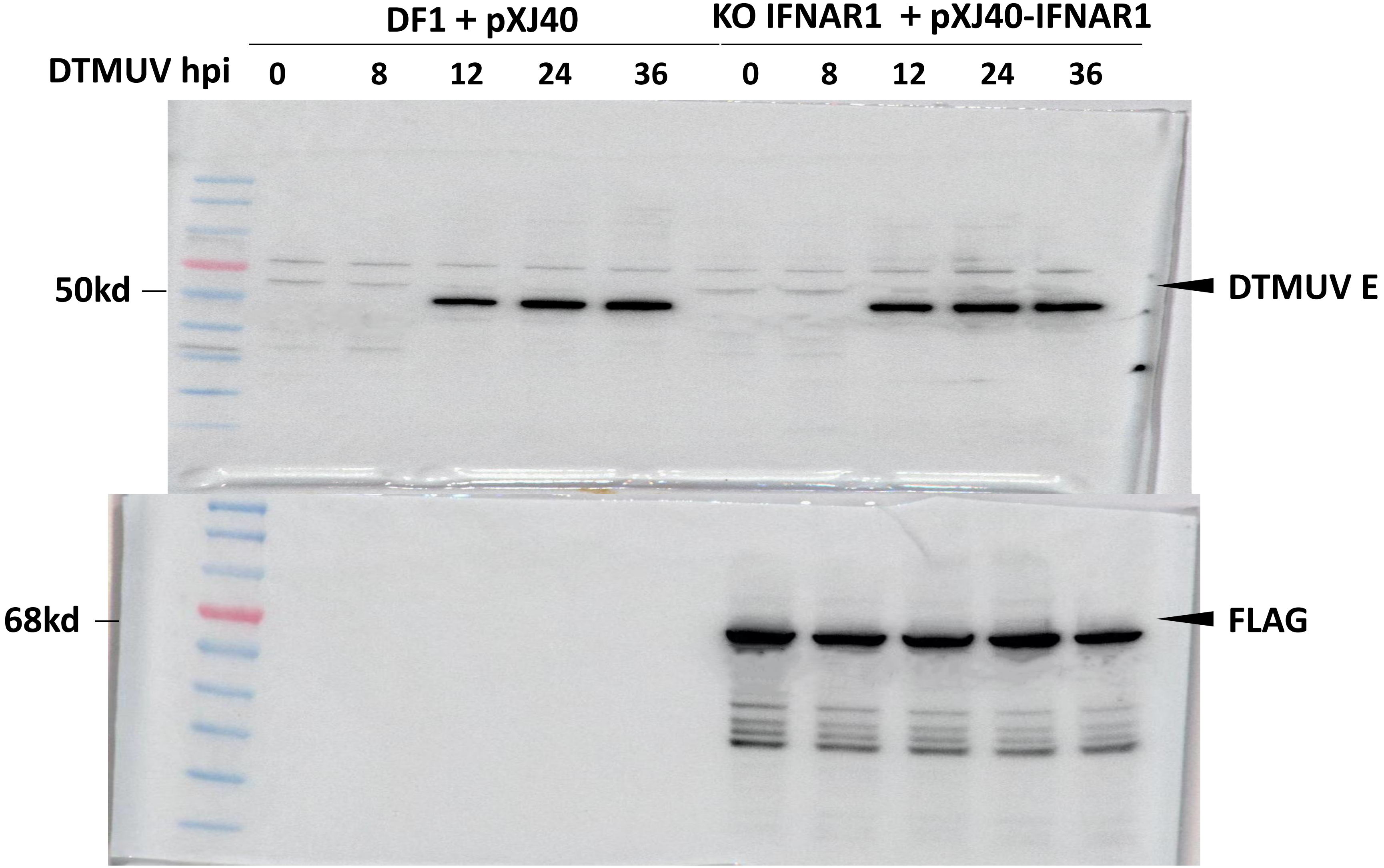

DF1 + pXJ40
KO IFNAR1 + pXJ40-IFNAR1
DTMUV hpi
0
8
12
24
36
0
8
12
24
36
50kd
DTMUV E
68kd
FLAG

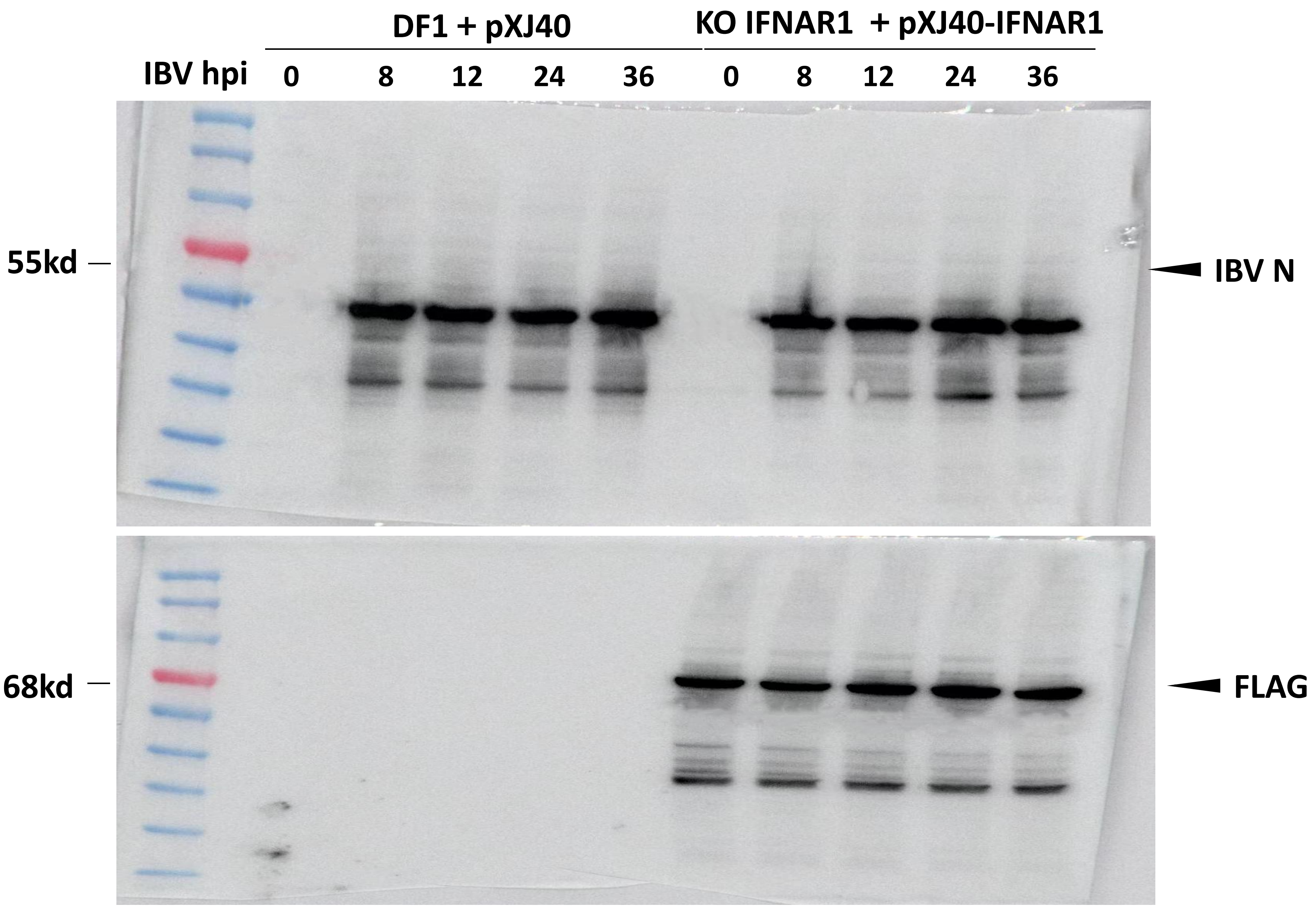
DF1 + pXJ40
KO IFNAR1 + pXJ40-IFNAR1
IBV hpi
0
8
12
24
36
0
8
12
24
36
55kd
IBV N
68kd
FLAG

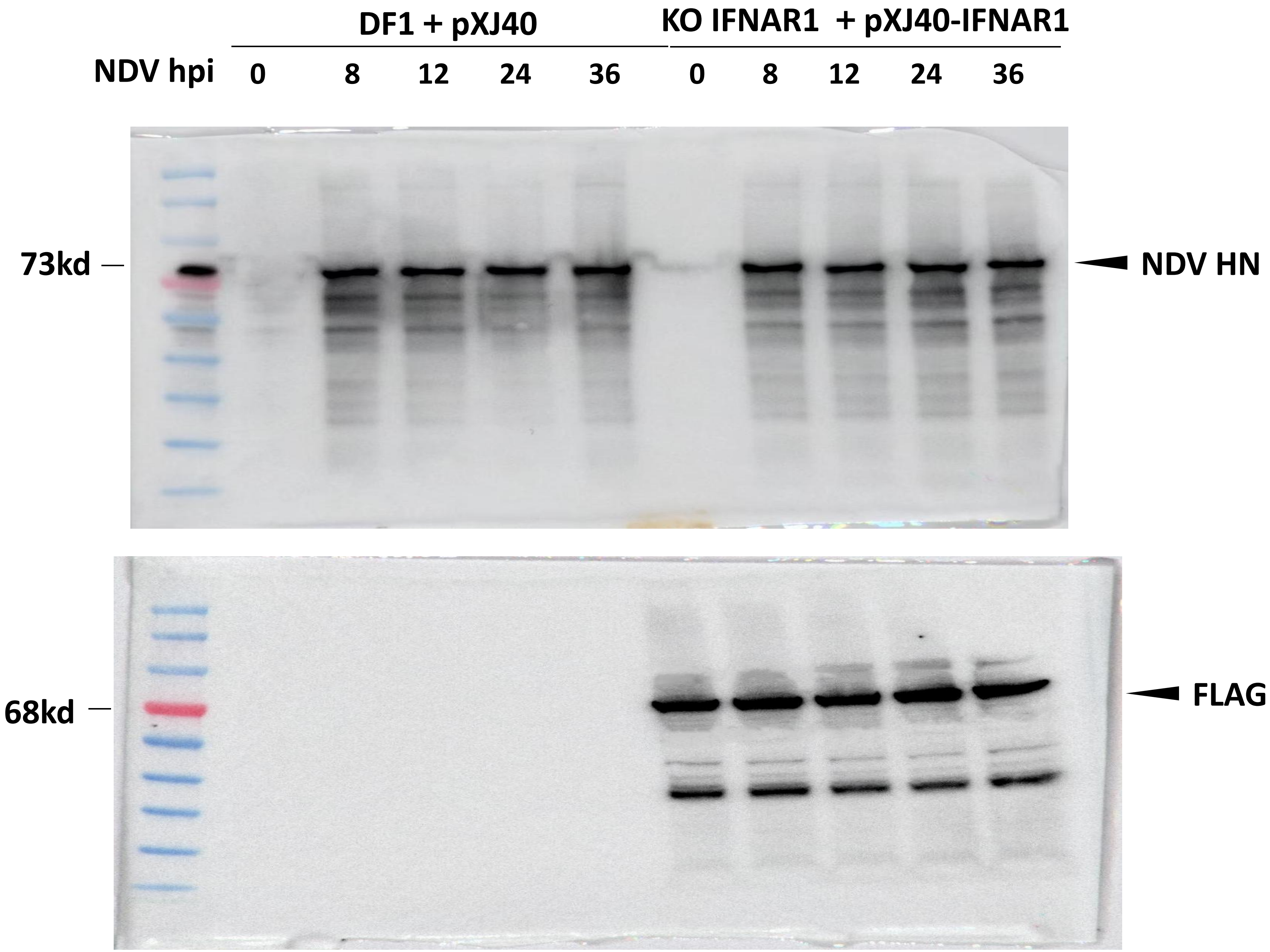
DF1 + pXJ40
KO IFNAR1 + pXJ40-IFNAR1
NDV hpi
0
8
12
24
36
0
8
12
24
36
73kd
NDV HN
68kd
FLAG
