# Supplementary figures and images for: Construction and Transcriptomic Study of Chicken IFNAR1-Knockout Cell Line Reveals the Essential Roles of Cell Growth- and Apoptosis-Related Pathways in Duck Tembusu Virus Infection
Source: Viruses. 2022 Oct 9;14(10):2225. doi: 10.3390/v14102225 (PMC9611459; doi:10.3390/v14102225)

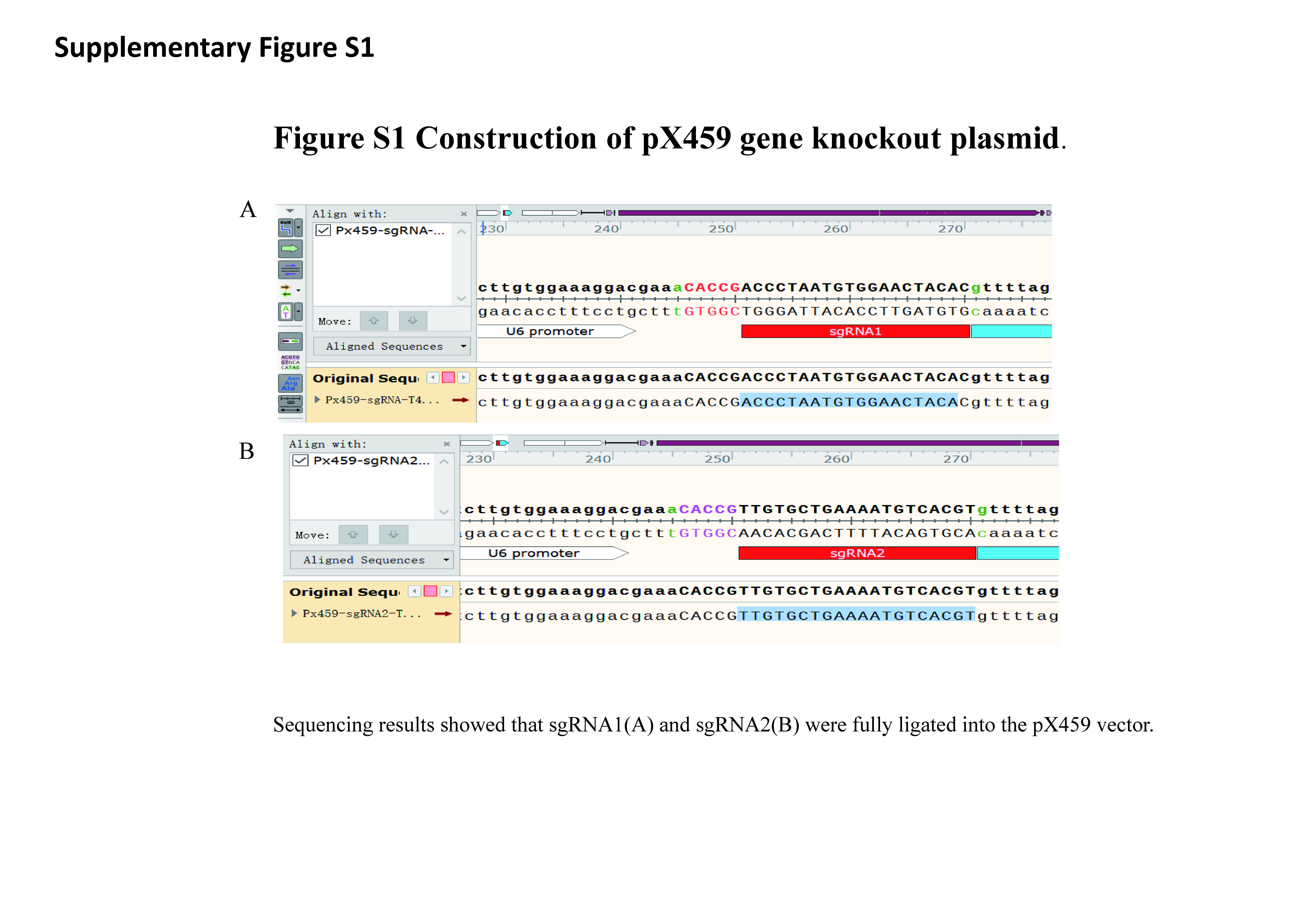

Supplement: Supplementary file 1 [file viruses-14-02225-s001.zip › supplementary/FIGURE S1.tif]

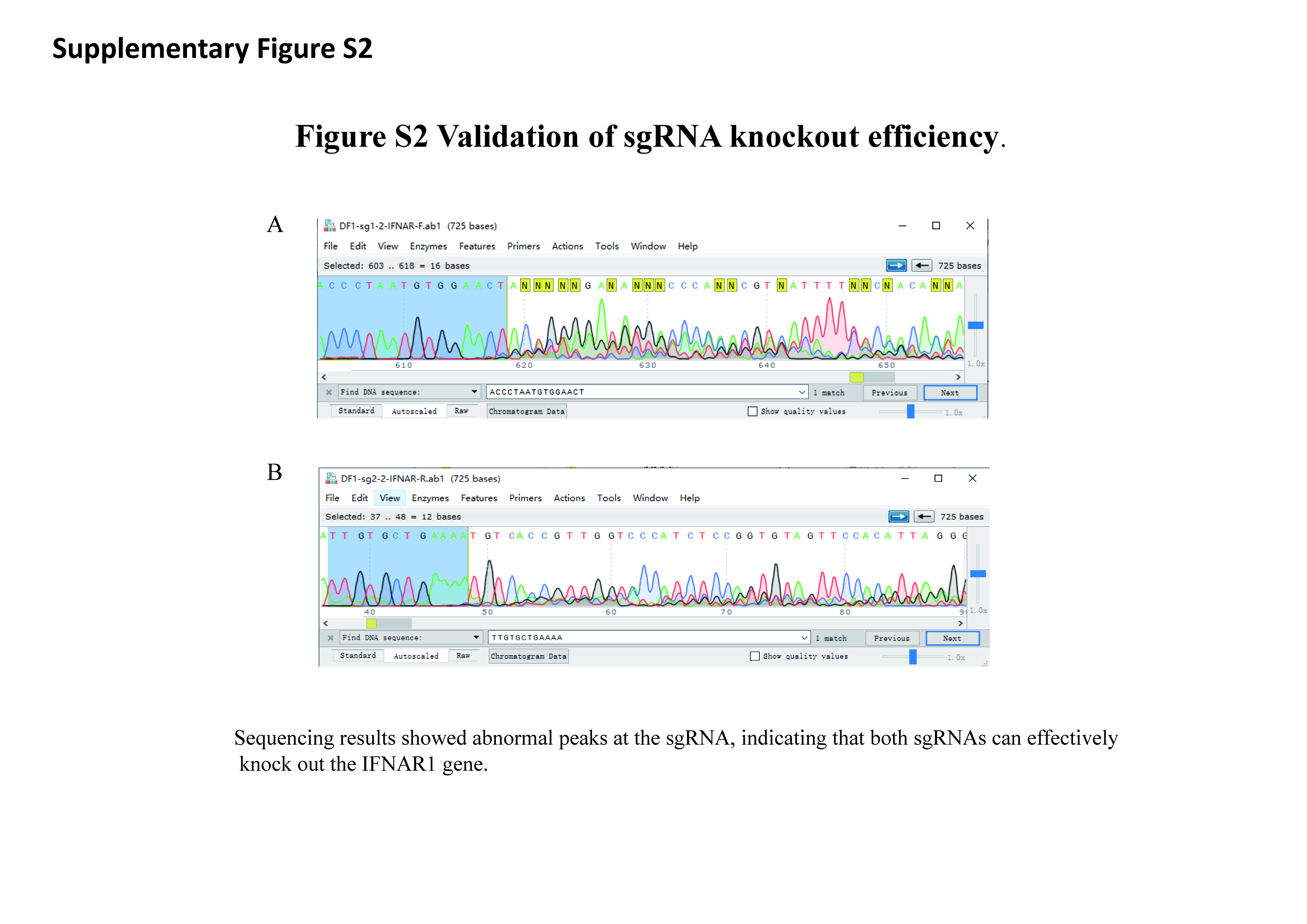

Supplement: Supplementary file 1 [file viruses-14-02225-s001.zip › supplementary/FIGURE S2.tif]

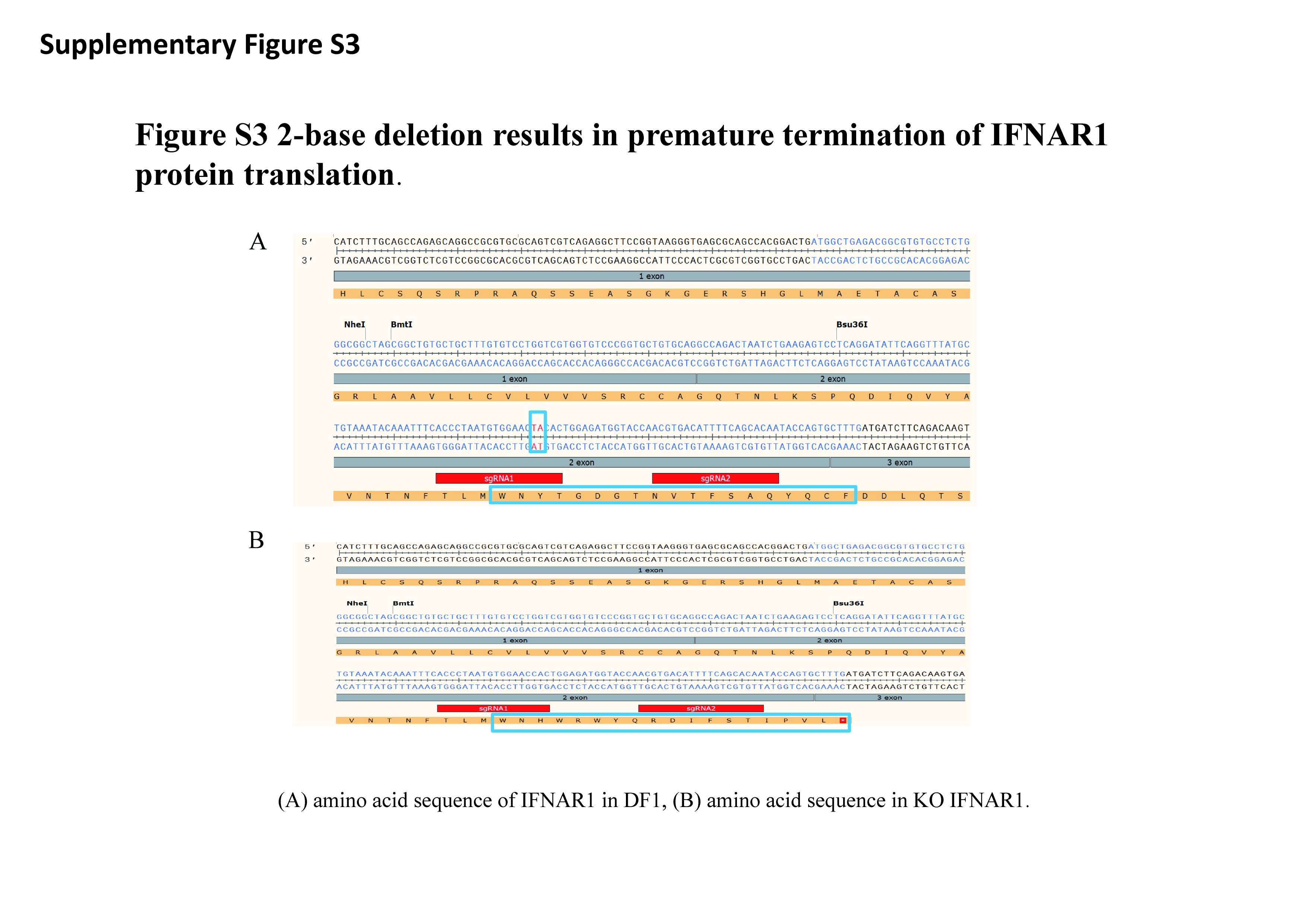

Supplement: Supplementary file 1 [file viruses-14-02225-s001.zip › supplementary/FIGURE S3.tif]

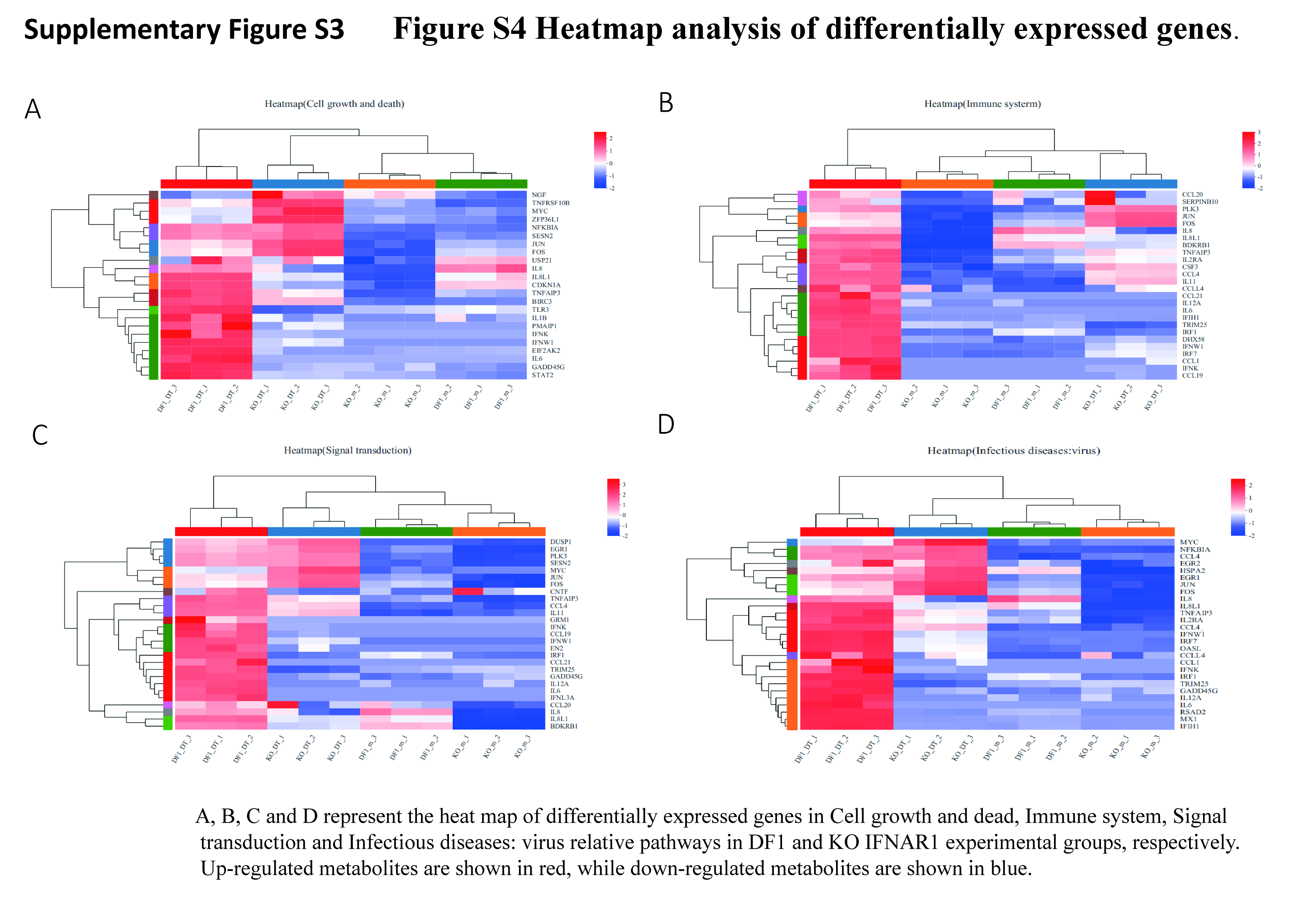

Supplement: Supplementary file 1 [file viruses-14-02225-s001.zip › supplementary/FIGURE S4.tif]

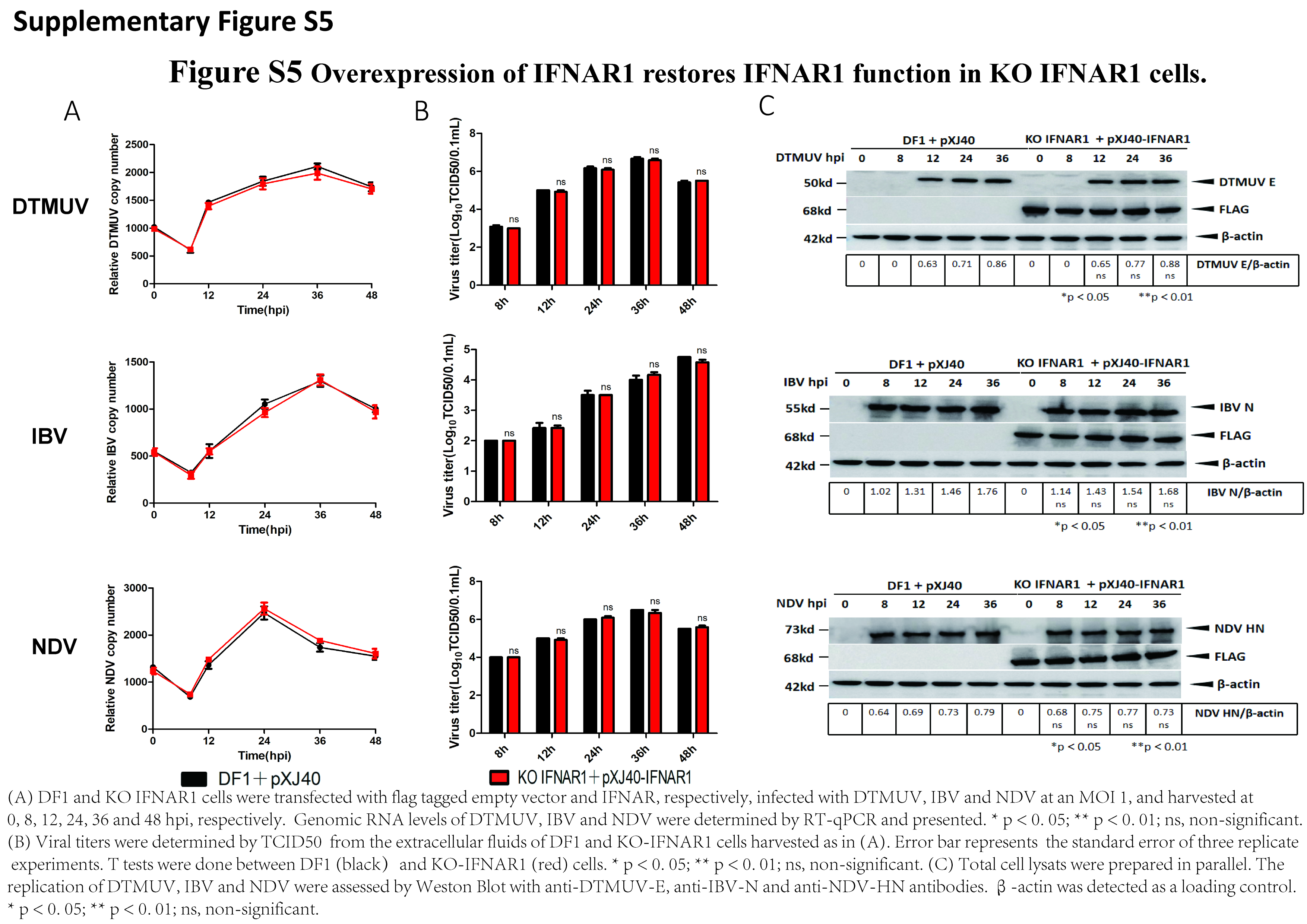

Supplement: Supplementary file 1 [file viruses-14-02225-s001.zip › supplementary/FIGURE S5.tif]
